# Elevated circulating amyloid concentrations in obesity and diabetes promote vascular dysfunction

**DOI:** 10.1172/JCI122237

**Published:** 2020-06-29

**Authors:** Paul J. Meakin, Bethany M. Coull, Zofia Tuharska, Christopher McCaffery, Ioannis Akoumianakis, Charalambos Antoniades, Jane Brown, Kathryn J. Griffin, Fiona Platt, Claire H. Ozber, Nadira Y. Yuldasheva, Natallia Makava, Anna Skromna, Alan Prescott, Alison D. McNeilly, Moneeza Siddiqui, Colin N.A. Palmer, Faisel Khan, Michael L.J. Ashford

**Affiliations:** 1 Division of Systems Medicine, School of Medicine, Ninewells Hospital and Medical School, Dundee, United Kingdom.; 2 Discovery and Translational Science Department, Leeds Institute of Cardiovascular and Metabolic Medicine, University of Leeds, Leeds, United Kingdom.; 3 Cardiovascular Medicine Division, Level 6 West Wing, John Radcliffe Hospital, Headington, Oxford, United Kingdom.; 4 School of Life Sciences, University of Dundee, Dundee, United Kingdom.; 5 Division of Population Health & Genomics, School of Medicine, Ninewells Hospital and Medical School, Dundee, United Kingdom.

**Keywords:** Endocrinology, Vascular Biology, Nitric oxide, Obesity, endothelial cells

## Abstract

Diabetes, obesity, and Alzheimer’s disease (AD) are associated with vascular complications and impaired nitric oxide (NO) production. Furthermore, increased β-site amyloid precursor protein–cleaving (APP-cleaving) enzyme 1 (BACE1), APP, and β-amyloid (Aβ) are linked with vascular disease development and increased BACE1 and Aβ accompany hyperglycemia and hyperlipidemia. However, the causal relationship between obesity and diabetes, increased Aβ, and vascular dysfunction is unclear. We report that diet-induced obesity (DIO) in mice increased plasma and vascular Aβ42 that correlated with decreased NO bioavailability, endothelial dysfunction, and increased blood pressure. Genetic or pharmacological reduction of BACE1 activity and Aβ42 prevented and reversed, respectively, these outcomes. In contrast, expression of human mutant APP in mice or Aβ42 infusion into control diet–fed mice to mimic obese levels impaired NO production, vascular relaxation, and raised blood pressure. In humans, increased plasma Aβ42 correlated with diabetes and endothelial dysfunction. Mechanistically, higher Aβ42 reduced endothelial NO synthase (eNOS), cyclic GMP (cGMP), and protein kinase G (PKG) activity independently of diet, whereas endothelin-1 was increased by diet and Aβ42. Lowering Aβ42 reversed the DIO deficit in the eNOS/cGMP/PKG pathway and decreased endothelin-1. Our findings suggest that BACE1 inhibitors may have therapeutic value in the treatment of vascular disease associated with diabetes.

## Graphical abstract

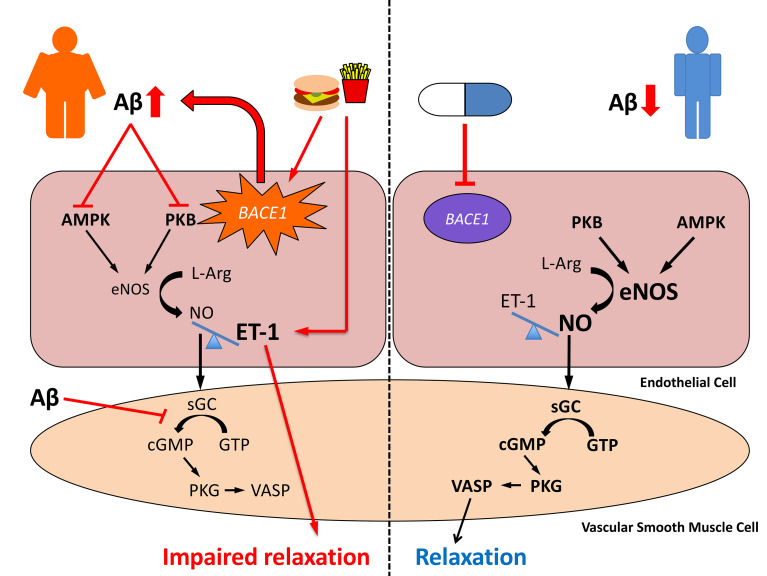

## Introduction

There is increasing evidence that links vascular disease and endothelial dysfunction (as found in obesity, diabetes, and hypercholesterolemia; i.e., metabolic syndrome) with Alzheimer’s disease (AD) and cognitive impairment, through many shared risk factors (1). Indeed, cognitive decline and AD are associated with cerebrovascular and coronary atherosclerosis in humans, and people with AD exhibit pathological signs of vascular impairment in cerebral and peripheral vessels, indicating some commonality between peripheral and central disease mechanisms (2). Moreover, vascular dysfunction occurs early in AD progression, often reported decades before the development of clinical symptoms of dementia (3). Recent work has demonstrated an interesting connection between one of the early biochemical events associated with AD and cerebral blood vessel dysfunction. The proteolytic cleavage of amyloid precursor protein (APP) by the enzyme, β-site APP-cleaving enzyme 1 (BACE1), is the rate-limiting step and one of the main drivers for production of β-amyloid (Aβ) peptides. Excessive accumulation of Aβ results in aggregation into amyloid plaques, a hallmark pathology of AD. Increased protein expression of APP, BACE1, and raised levels of Aβ peptides (Aβ40 and Aβ42) have been demonstrated in cerebral blood vessels in humans with AD and are detectable in experimental models of AD before the appearance of AD pathology and cognitive decline (4–7). Therefore, increased APP processing and Aβ production may be directly linked to endothelial dysfunction in cerebral and peripheral blood vessels. However, the role of APP processing and Aβ production in endothelial dysfunction in obesity and diabetes is not clear.

Endothelial dysfunction is frequently observed in experimental models of diabetes (8, 9) and is characterized, in part, by impaired nitric oxide–mediated (NO-mediated) relaxation. Furthermore, diabetes is commonly associated with microvascular and macrovascular complications, including ischemic heart disease, peripheral vascular disease, atherosclerosis, and congestive heart failure, accounting for up to 80% of the excess mortality in patients with diabetes (10). In a clinical setting endothelial dysfunction, which starts early in diabetes development, is strongly associated with adverse outcomes (11) and contributes to the development of insulin resistance, microangiopathy (12), and atherosclerosis (13). Importantly, impaired endothelium-dependent vasodilation is measurable before any morphological changes are detected in the vessel wall (14), indicating that reduced NO production and/or activity may be an important early event in vascular pathogenesis.

Aβ peptides at high concentrations are toxic to brain and peripheral endothelial cells, causing cellular damage, enhanced vasoconstriction, and impairment of endothelium-dependent relaxation, thereby promoting atherosclerosis and vascular disease (15). These actions are associated with reduced glucose delivery, increased hypoxia, and oxidative stress in the neurovascular unit and are significant contributors to the pathology that defines neurodegeneration and AD. In addition, APP and Aβ are detected in human carotid plaques and atherosclerotic aortas (16), and overexpression of APP accelerates (17), whereas deletion partially protects against (18), the development of aortic atherosclerosis in *APOE^–/–^* mice. Ex vivo and in vitro studies have demonstrated that soluble Aβ peptides reduce endothelial NO production, potentially by inhibition of endothelial NO synthase (eNOS) phosphorylation at Ser^1177^ (19, 20). However, these studies used excessively high (~1 μM) concentrations of Aβ peptides, whereas reported in vivo levels are significantly lower (21, 22) and vary from approximately 5 to 800 pg/mL (~1–200 pM) for Aβ42 and 35 to 700 pg/mL for Aβ40 (~9–180 pM) in plasma. Interestingly, eNOS modulates amyloid processing, as reduction or loss of eNOS is associated with increased expression of APP and BACE1 and the production of Aβ peptides, whereas exogenous NO reduces APP, BACE1, and Aβ levels in cerebral microvessels (23). Thus, there appears to be an interesting reciprocal connection between BACE1, APP processing, Aβ levels, and NO bioavailability. Hyperglycemia and hyperlipidemia increase BACE1 activity and Aβ peptide levels in tissues and plasma (24, 25), linking key metabolic disease markers with increased amyloid processing. Consequently, we hypothesized that the development of type 2 diabetes (T2D) and/or obesity elevates circulating Aβ levels, which in turn drives vascular dysfunction. We tested this hypothesis in 3 ways: firstly, by reducing BACE1 activity genetically and pharmacologically in mice and ascertaining how this modified diet-induced endothelial dysfunction; secondly, by increasing plasma Aβ levels, indirectly through overexpression of mutant human APP genes and directly by Aβ peptide infusion, to promote vascular dysfunction in mice; and thirdly, by cross-sectionally examining the association between plasma Aβ levels and endothelial function in patients with T2D.

## Results

### BACE1 expression and activity in the vasculature.

**Figure 1:**
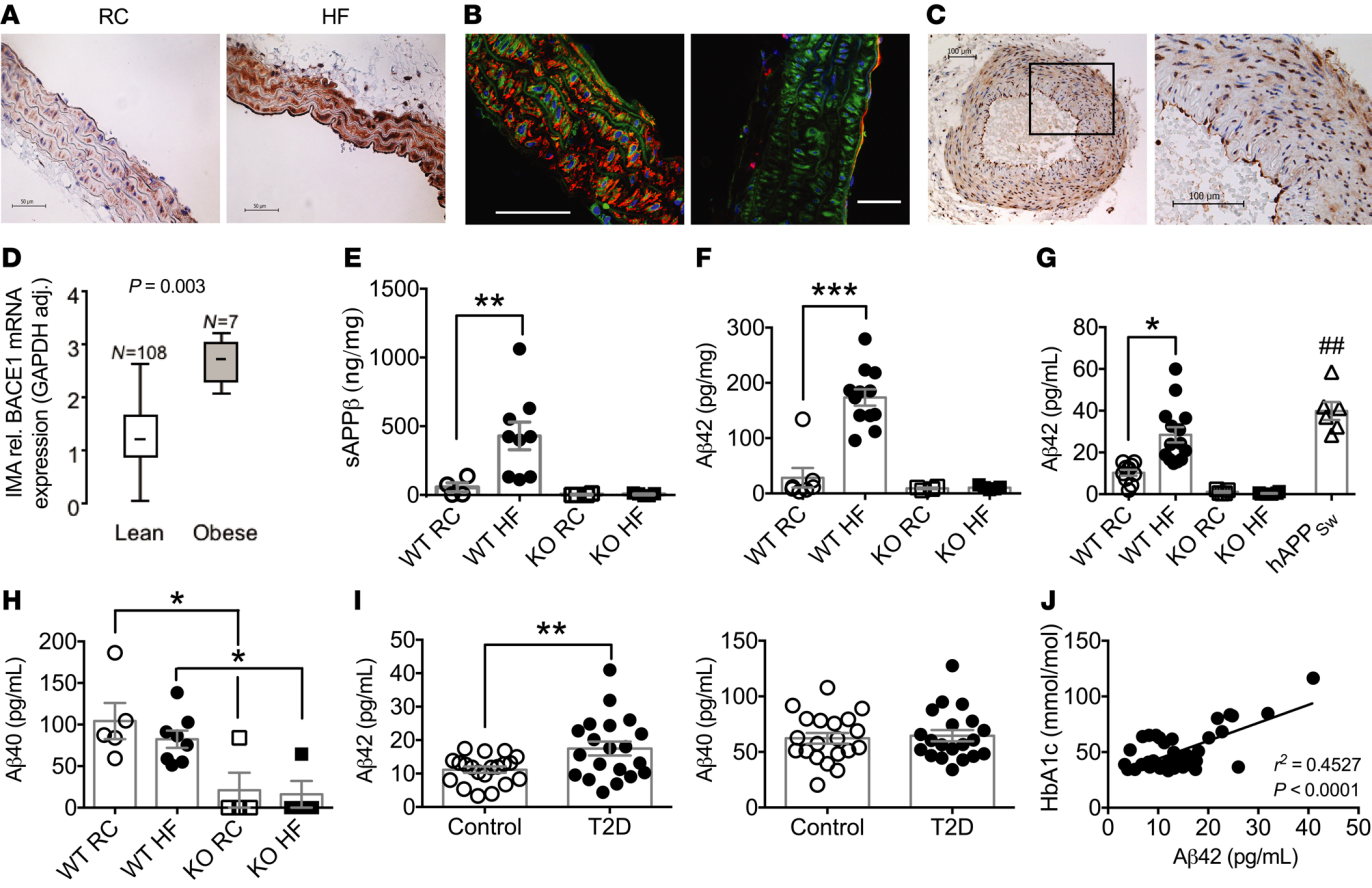
BACE1 vascular expression and activity and plasma levels of Aβ in mice and humans. (**A**) Immunohistochemical staining for BACE1 (brown) showing representative sections from aortas of RC-fed and HF-fed (DIO) WT mice. (**B**) Confocal images of DIO mouse aortas stained for BACE1 (green) and SMA (left) or CD31 (right), respectively (red). Scale bars: 50 μm. (**C**) Immunohistochemical staining for BACE1 in human nonatherosclerotic temporal artery, with a section (rectangle) at higher magnification. (**D**) BACE1 mRNA expression in internal mammary arteries from lean and obese individuals (Mann-Whitney *U* test). HF feeding increases BACE1 activity in WT mouse aorta, as determined by sAPPβ (**E**) and Aβ42 (**F**) levels, with *BACE1*-KO aortas showing negligible peptide levels (*n* = 4–12). (**G**) Plasma Aβ42 levels are increased in DIO mice to RC-fed *hAPP_Sw_* mouse levels, with insignificant plasma Aβ42 in RC- or HF-fed *BACE1*-KO mice (*n* = 6–14). (**H**) Plasma Aβ40 levels of RC-fed and DIO mice and RC- and HF-fed *BACE1*-KO mice (*n* = 6–14). (**I**) Plasma Aβ42 and Aβ40 levels in control (*n* = 20) and obese individuals with type 2 diabetes (T2D) (*n* = 20). (**J**) Linear regression between plasma Aβ42 and HbA1c in control and obese individuals with T2D (*P* < 0.001). Data presented as means ± SEM for all figures except **D**, where SD given. *P < 0.05; **P < 0.01; ^##^*P* < 0.01; ***P < 0.001 by 2-way ANOVA with Tukey’s multiple-comparisons test (**E**–**H**) or 2-tailed unpaired Student’s *t* test (**G** and **I**).

BACE1 protein was detected in vascular tissue (aorta) from regular chow–fed (RC-fed) wild-type (WT) mice, with expression increased (Figure 1A) in mice made obese by high-fat (HF) diet for 20 weeks (diet-induced obese [DIO] mice; Table 1 and Supplemental Figure 1A; supplemental material available online with this article; https://doi.org/10.1172/JCI122237DS1). BACE1 protein was localized (Figure 1B) to endothelial and vascular smooth muscle cells (VSMCs). BACE1 protein expression was detectable in control (nonobese/diabetic) human temporal arteries (Figure 1C) and the expression of *BACE1* mRNA in human internal mammary artery was higher in obese (body mass index [BMI] ≥ 35 kg/m^2^) compared with lean individuals (Figure 1D), although no difference in *BACE1* mRNA expression was observed when correlated with insulin resistance or diabetes (Supplemental Figure 2 and Supplemental Table 1). BACE1 activity, as measured by soluble APPβ (sAPPβ) levels, in the aorta of DIO mice was increased compared with age-matched RC-fed controls or BACE1-knockout (*BACE1*-KO) mice on either diet (Figure 1E; main effect of diet, *P* < 0.05), with a corresponding increase in DIO mouse aorta of the sequential γ-secretase cleavage product, Aβ42 (Figure 1F; diet × genotype, *P* < 0.001). Levels of sAPPβ and Aβ42 were barely detectable in the aortas of *BACE1*-KO mice, on either RC or HF diet (Figure 1, E and F).

### Aβ40 and Aβ42 plasma levels in mice and humans.

Plasma levels of Aβ42 were increased in DIO mice in comparison with age-matched RC-fed controls, to levels that were not significantly different from that obtained in RC-fed AD transgenic mice expressing human APP with the Swedish mutation (*hAPP_Sw_* mice), with *BACE1*-KO mice displaying very low plasma Aβ42 levels (Figure 1G; diet × genotype, *P* < 0.01). Plasma Aβ40 levels were also reduced in *BACE1*-KO mice compared with age-matched RC-fed and DIO mice; however, plasma Aβ40 levels in WT mice were not sensitive to the HF diet (Figure 1H; main effect of genotype, *P* < 0.001). In agreement with the results for DIO mice, we found that human plasma Aβ42, but not Aβ40, levels (Human Study 1), were increased (Figure 1I) in T2D patients (*n* = 20) versus a control (*n* = 20) group matched for age (61.2 ± 12.5 and 67.2 ± 10.4 years, respectively) and sex (male/female = 9:11 and 8:12, respectively) and show a significant linear relationship with glycated hemoglobin (HbA1c) (Figure 1J), with mean values for HbA1c of 63.8 ± 4.2 and 38.0 ± 0.7 mmol/mol (*P* = 0.0001), respectively. BMI was greater in patients with T2D (32.3 ± 1.7 and 26.7 ± 0.9 kg/m^2^, *P* = 0.007). There was no relationship between Aβ42 levels and age in this cohort, tested by linear regression (*r*^2^ = 0.014, *P* = 0.31).

### Genetic reduction of BACE1 protects against HF diet–induced vascular dysfunction.

**Figure 2:**
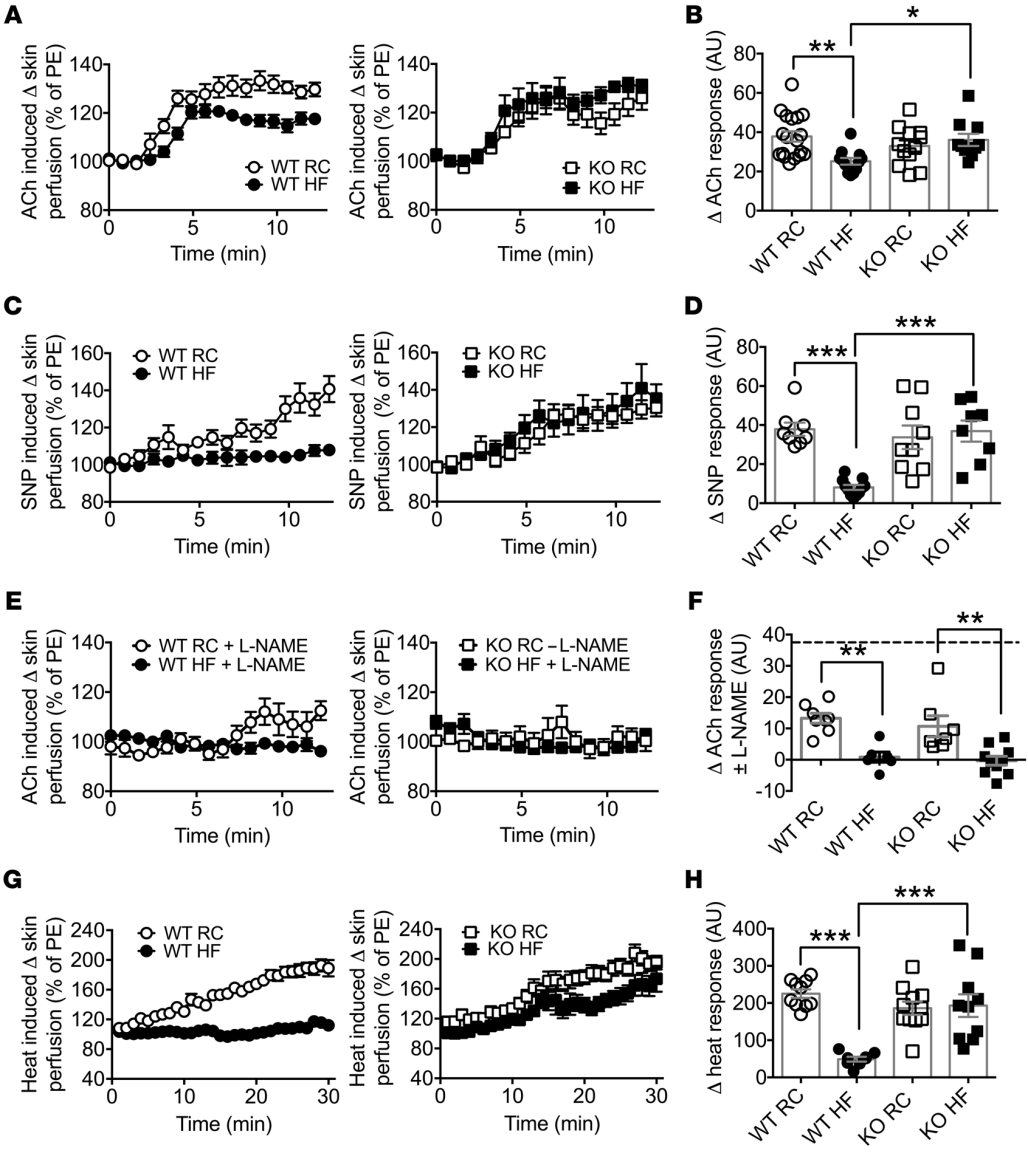
Loss of BACE1 prevents HF feeding–induced endothelial and vascular smooth muscle dysfunction. (**A**) Endothelium-dependent microvascular responses induced by ACh in RC-fed WT, DIO (20-week HF-fed) mice, and RC- and HF-fed *BACE1*-KO mice (*n* = 10–21). PE, phenylephrine. (**B**) Quantitative analysis of ACh response from **A**. (**C**) Endothelium-independent responses induced by SNP in RC-WT, DIO, and *BACE1*-KO mice (*n* = 8–9). (**D**) Quantitative analysis of SNP responses from **C**. (**E**) Microvascular responses induced by ACh in the presence of L-NAME in RC-fed WT, DIO, and RC- and HF-fed WT and *BACE1*-KO mice (*n* = 6–10). (**F**) Quantitative analysis of ACh responses from **E**. The broken line denotes the RC-fed WT ACh response. (**G**) Microvascular responses to localized heating in RC-fed WT, DIO, and RC- and HF-fed *BACE1*-KO mice (*n* = 9–11). (**H**) Quantitative analysis of heating responses from **G**. Data are means ± SEM. *P < 0.05; **P < 0.01; ***P < 0.001 by 2-way ANOVA with Tukey’s multiple-comparisons test.

The role of BACE1 activity in endothelium-dependent and -independent vascular responses was examined in the microvasculature of RC-fed WT, DIO, and RC- and HF-fed *BACE1*-KO mice. We also measured body weight, fasted blood glucose, and glucose and insulin sensitivity in the mice, as these parameters are sensitive to BACE1 levels and activity (26, 27). DIO mice, which developed obesity with hyperglycemia and insulin resistance (Supplemental Figure 1, B–D, and Table 1) showed impaired endothelium-dependent vasodilation induced by iontophoresis of acetylcholine (ACh) (Figure 2, A and B) and endothelium-independent vasodilation by iontophoresis of the NO donor sodium nitroprusside (SNP) (Figure 2, C and D), compared with RC-fed WT mice. In contrast, *BACE1*-KO mice (RC- or HF-fed) displayed no impairment in endothelium-dependent (Figure 2, A and B; diet × genotype, *P* < 0.01) or endothelium-independent vasodilation by diet (Figure 2, C and D; diet × genotype, *P* < 0.001). To establish the relative role of endothelium-derived NO in the ACh-mediated vascular responses, mice were pretreated with the NO synthase inhibitor *N*^G^-nitro-L-arginine methyl ester hydrochloride (L-NAME). Administration of L-NAME blunted vascular responses to ACh in RC-fed WT, DIO, and RC- and HF-fed *BACE1*-KO mice (Figure 2, E and F; main effect of diet, *P* < 0.001), demonstrating that a major proportion of the ACh-induced vascular response is NO dependent in both WT and *BACE1*-KO mice. Thus, in contrast to WT mice, *BACE1*-KO mice retain normal NO-mediated vasodilation after chronic HF feeding.

To determine the contribution of BACE1 activity and dietary challenge to the maximum dilatory capacity of the vasculature, the response to localized heating was determined (Figure 2, G and H). Although DIO mice displayed a significantly impaired vasodilator response to heat compared with RC-fed WT mice, RC- and HF-fed *BACE1*-KO mice exhibited a maximal vasodilator response to heating that was comparable to WT mice fed an RC diet (diet × genotype, *P* < 0.001). Collectively, these results clearly implicate BACE1 activity in the control of endothelium-dependent and endothelium-independent vascular relaxation of HF-fed mice in response to ACh, SNP, and raised skin temperature.

### Enhanced amyloidogenic processing impairs vascular function in RC-fed mice.

**Figure 3:**
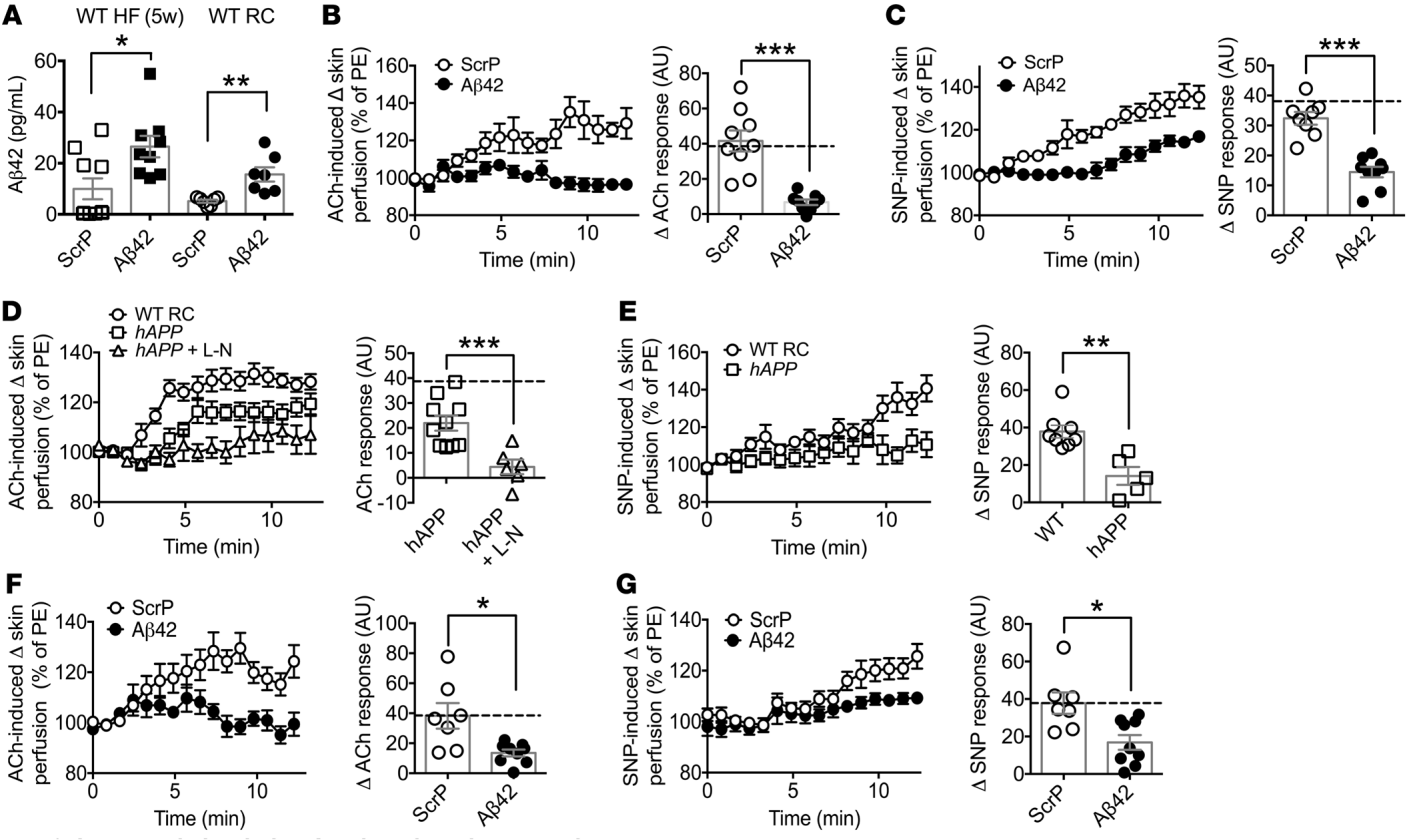
Increased circulating Aβ42 impairs microvascular function. (**A**) Plasma Aβ42 levels in HF-fed (5 weeks) and RC-fed WT mice after 4 weeks of Aβ42 or ScrP infusion (*n* = 7–10). (**B**) Endothelium-dependent microvascular responses induced by ACh in ScrP- and Aβ42-treated HF-fed (5 weeks) WT mice (*n* = 9). Quantitative analysis of ACh responses for ScrP- and Aβ42-treated mice. The broken line denotes the RC-fed WT ACh response. PE, phenylephrine. (**C**) Endothelium-independent responses induced by SNP in ScrP- and Aβ42-treated HF-fed (5 weeks) WT mice (*n* = 8–9). Quantitative analysis of SNP responses for ScrP- and Aβ42-treated mice. The broken line denotes the RC-fed WT SNP response. (**D**) Microvascular responses induced by ACh in RC-fed WT and *hAPPSw* mice and *hAPPSw* mice with L-NAME (L-N) (*n* = 6–16). Quantitative analysis of ACh responses for *hAPPSw* mice in the absence and presence of L-NAME. The broken line denotes the RC-fed WT ACh response. (**E**) Endothelium-independent responses induced by SNP in RC-fed WT and *hAPPSw* mice (*n* = 5–8). Quantitative analysis of SNP responses. (**F**) Endothelium-dependent microvascular responses induced by ACh in ScrP- and Aβ42-treated RC-fed WT mice (*n* = 7–9). Quantitative analysis of ACh responses for ScrP- and Aβ42-treated mice. The broken line denotes the RC-fed WT ACh response. (**G**) Endothelium-independent responses induced by SNP in ScrP- and Aβ42-treated RC-fed WT mice (*n* = 7–9). Quantitative analysis of SNP responses for ScrP- and Aβ42-treated mice. The broken line denotes the RC-fed WT SNP response. Data are means ± SEM. *P < 0.05; **P < 0.01; ***P < 0.001 by 2-tailed unpaired Student’s *t* test.

The primary substrate identified for BACE1 is APP, with HF feeding increasing BACE1 activity and enhancing APP cleavage that increases Aβ peptide levels (28). We found that chronic HF feeding leading to obesity increased Aβ42, but not Aβ40, levels in plasma (Figure 1, G and H). To address further the role of Aβ42 in vascular dysfunction, we infused WT mice with a physiological dose of mouse Aβ42, or equivalent dose of scrambled peptide (ScrP) as control, either early in the HF feeding regime (infusion begun after 1 week of HF diet), at a stage when vascular dysfunction is not evident (Supplemental Figure 3) and plasma Aβ42 levels remain low, or at an equivalent age in mice maintained on RC diet. Total mean Aβ42 plasma levels in the Aβ42-infused, but not ScrP-infused, 5-week HF- or RC-fed mice, reached quantities comparable to that obtained for DIO mice (i.e., after 20 weeks of HF feeding) (Figure 3A and Figure 1G). The elevated plasma Aβ42 caused impaired ACh-induced vasodilation, in contrast to the ScrP-treated mice, which retained normal responses to ACh (Figure 3B). Similarly, the presence of raised plasma Aβ42 impaired SNP-mediated vasodilation, compared with ScrP-treated mice in 5-week HF-fed mice (Figure 3C). These data led us to examine whether raised physiological or pathophysiological levels of Aβ42 were the primary driver for the compromised vascular responses associated with the HF diet.

Consequently, we utilized 2 models where circulating Aβ42 levels are increased, independently of HF diet. First, vascular responses were measured in *hAPP_Sw_* mice (29) on RC diet. Overexpression of the *hAPP_Sw_* form in these mice significantly increased total plasma Aβ42 levels (Figure 1G) to levels comparable to DIO mice. The elevated plasma Aβ42 impaired ACh-induced vasodilation in the *hAPP_Sw_* mice, which was further reduced in the presence of L-NAME by a similar amount compared with responses for WT RC-fed mice (80% vs. 65% reduction), demonstrating that the ACh response, although diminished, remained predominantly NO dependent (Figure 3D). Furthermore, the SNP-induced vasodilation was also impaired in *hAPP_Sw_* mice, indicating that enhanced Aβ production also results in the development of VSMC dysfunction (Figure 3E). Next, we infused Aβ42 or ScrP into WT mice maintained on RC diet for 28 days (Figure 3A). This increased plasma Aβ42 had no effect on body weight or fasted blood glucose levels (Table 2) but reduced vascular responses to ACh (Figure 3F) and SNP (Figure 3G), indicating that increased plasma Aβ42 is responsible for the inhibition of NO-mediated vasodilation, independent of dietary influence.

### BACE1 inhibition reverses vascular dysfunction.

**Figure 4:**
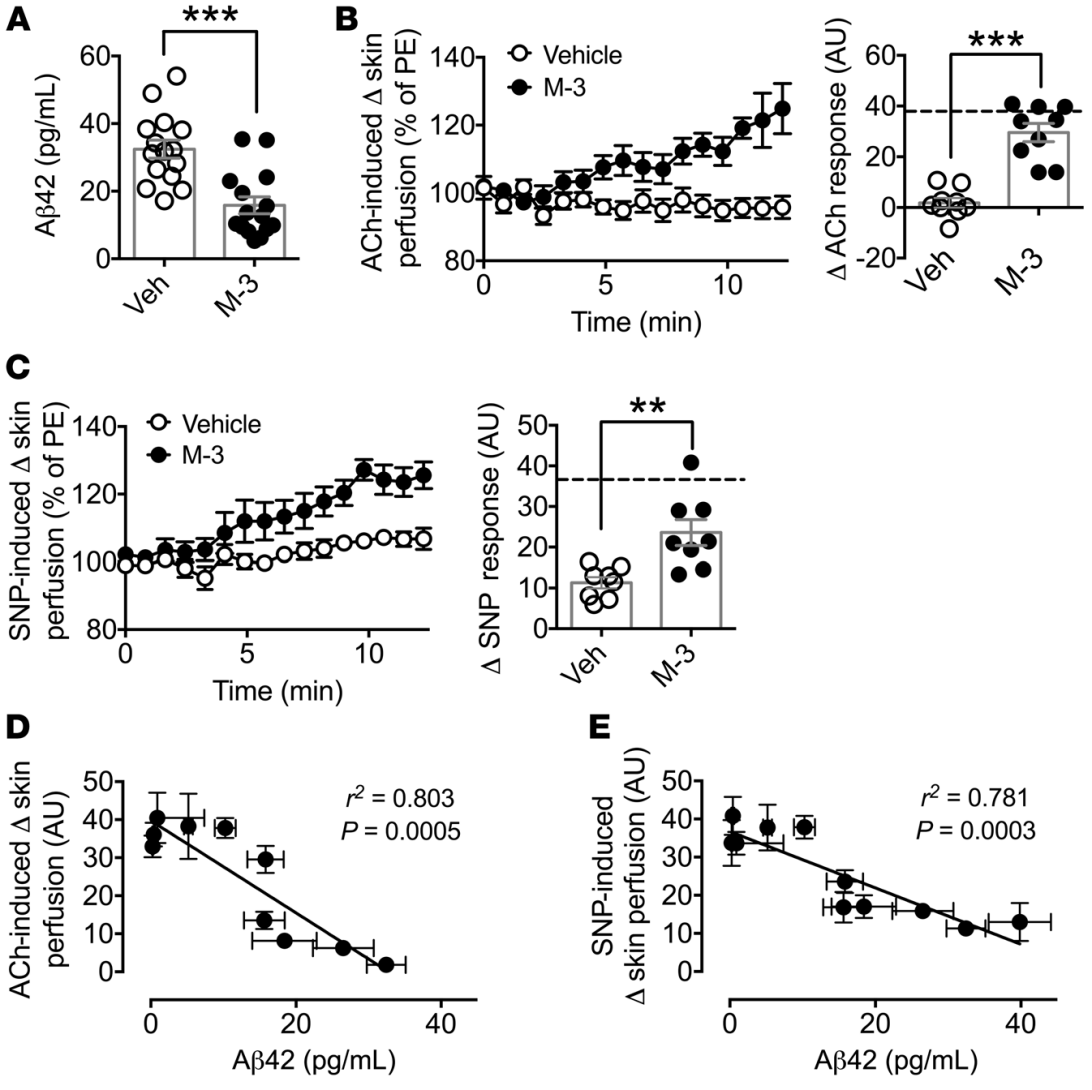
M-3 recovers microvascular function in DIO mice and plasma Aβ42 correlates with microvascular responsiveness. (**A**) Plasma Aβ42 levels of DIO mice treated with M-3 or vehicle (*n* = 8). (**B**) Endothelium-dependent microvascular responses induced by ACh in vehicle- and M-3–treated DIO mice (*n* = 9). Quantitative analysis of ACh responses for vehicle- and M-3–treated mice. The broken line denotes the RC-fed WT ACh response. PE, phenylephrine. (**C**) Endothelium-independent responses induced by SNP in vehicle- and M-3–treated DIO mice (*n* = 8). Quantitative analysis of SNP responses for vehicle- and M-3–treated mice. The broken line denotes the RC-fed WT SNP response. Linear regression between plasma Aβ42 levels and ACh (**D**) and SNP (**E**) responses for experimental groups of mice. Data are means ± SEM. **P < 0.01, ***P < 0.001 by 2-tailed unpaired Student’s *t* test.

We subsequently examined whether reduction of the elevated Aβ42 levels by pharmacological intervention might be a potential means to reverse vascular dysfunction. First, a 4-week infusion of the BACE1 inhibitor M-3 reduced plasma Aβ42 levels (Figure 4A) and significantly improved ACh-induced vasodilation in DIO mice, with responses recovered to values similar to RC-fed WT mice (dashed line; Figure 4B). In addition, endothelium-independent (SNP) vasodilator responses were improved by the infusion of M-3 in DIO mice, although the mean response amplitude was lower than that of WT RC-fed mice (dashed line; Figure 4C). The treatment of DIO mice with M-3 also reduced their fasted blood glucose levels and lowered body weight (Table 2), as reported previously (30). Next, the effects of BACE1 inhibitor infusion were examined in a mouse model overexpressing human APP, the *hAPP23* mouse (31), which has higher expression of APP than *hAPP_Sw_*, and exhibits early cognitive impairment and extensive Aβ pathology (at 3–6 months) (32). *hAPP23* mice at 4 months of age, like *hAPP_Sw_*, displayed impaired vasodilation responses to ACh and SNP and these responses were significantly improved by the presence of M-3 (Supplemental Figure 4, A and B).

Taken together, these data demonstrate that pathophysiological levels (>20 pg/mL) of plasma Aβ42 impair vascular responses independently of the presence of obesity and/or diabetes. Furthermore, as HF feeding preferentially promotes Aβ42 production, the increased level of this amyloid peptide species may underlie the mechanism by which obesity results in vascular dysfunction. This is further highlighted when the magnitude of the ACh- (Figure 4D) or SNP-induced (Figure 4E) vasodilation responses for the experimental protocols reported here is plotted against plasma Aβ42 levels, giving inverse relationships, illustrating that decreased ACh and SNP responsiveness correlates with higher plasma Aβ42 levels. Consequently, targeting increased BACE1 activity to reduce the increased Aβ42 levels found in obesity- and diabetes-driven vascular disease may recover vascular function.

### Aβ42 suppresses NOx while enhancing ET-1 production.

**Figure 5:**
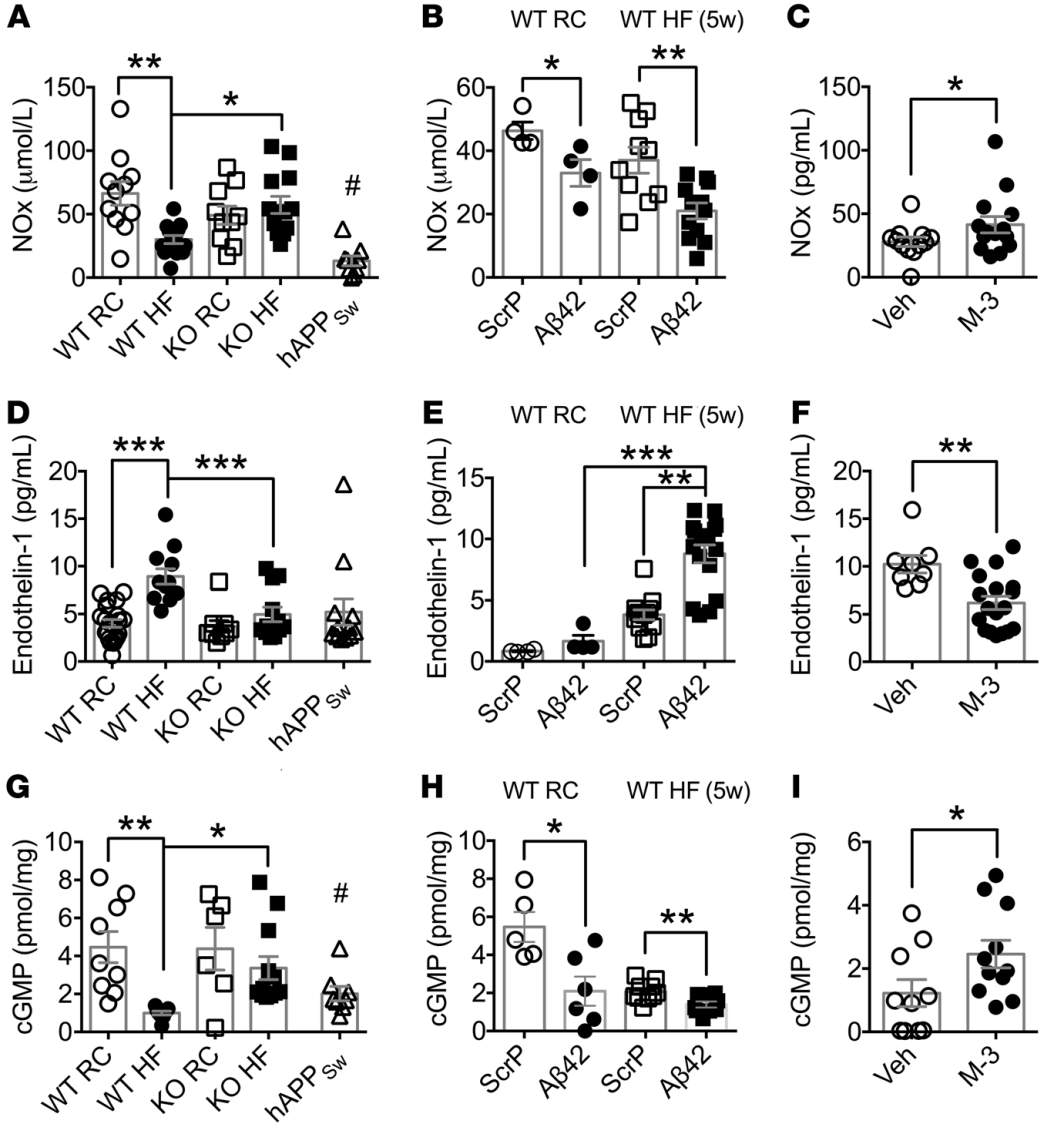
Plasma NOx, ET-1, and aortic cGMP are modified by BACE1 activity and Aβ42. (**A**) Plasma NOx levels in RC-fed WT, *hAPP_Sw_*, and *BACE1*-KO, DIO, and HF-fed *BACE1*-KO mice (*n* = 9–14). (**B**) Plasma NOx levels in RC-fed WT and HF-fed (5 weeks) mice after 4 weeks of Aβ42 or ScrP infusion (*n* = 4–12). (**C**) Plasma NOx levels in vehicle- and M-3–treated DIO mice (*n* = 12–14). (**D**) Plasma ET-1 levels in RC-fed WT, *hAPP_Sw_*, and *BACE1*-KO, DIO, and HF-fed *BACE1*-KO mice (*n* = 9–21). (**E**) Plasma ET-1 levels in RC-fed WT and HF-fed (5 weeks) mice after 4 weeks of Aβ42 or ScrP infusion (*n* = 4–12). (**F**) Plasma ET-1 levels in vehicle- and M-3–treated DIO mice (*n* = 8–18). (**G**) Aortic cGMP levels in RC-fed WT, *hAPP_Sw_*, and *BACE1*-KO, DIO, and HF-fed *BACE1*-KO mice (*n* = 6–12). (**H**) Aortic cGMP levels in HF-fed (5 weeks) mice after 4 weeks of Aβ42 or ScrP infusion (*n* = 9–10) and (**I**) in vehicle- and M-3–treated DIO mice (*n* = 10–11). Data are means ± SEM. *P < 0.05; **P < 0.01; ***P < 0.001 by 2-way ANOVA with Tukey’s multiple-comparisons test (**A**, **B**, **D**, **E**, **G**, and **H**) or 2-tailed unpaired Student’s *t* test (**C**, **F**, and **I**). ^#^*P* < 0.05 vs. RC-fed WT (**A** and **G**).

Excessive endothelin-1 (ET-1) production coupled with reduced NO levels results in a critical imbalance between these 2 potent vasomodulators and subsequently promotes vascular dysfunction. Accordingly, we set out to determine whether the raised levels of Aβ42 associated with our mouse models alters the amounts of NO and ET-1 and contributes to this outcome. Therefore, first we determined whether decreased bioavailability of NO aids the suppression of vasodilation in mice with increased plasma Aβ42. We found that the levels of total plasma NO (NOx) were diminished in WT mice by HF feeding, whereas NOx levels were unchanged in *BACE1*-KO mice on an RC or HF diet (Figure 5A; diet × genotype, *P* < 0.01). NOx was also found to be suppressed in *hAPP_Sw_* (Figure 5A) and *hAPP23* mice (Supplemental Figure 4C) on RC diet. Furthermore, RC-fed and short-term (5 weeks) HF-fed WT mice infused with Aβ42 (versus ScrP controls) also exhibited reduced NOx levels (Figure 5B; main effect of treatment, *P* < 0.01), whereas DIO mice given the BACE1 inhibitor M-3 displayed increased NOx levels (Figure 5C). *hAPP23* mice given M-3 also showed a trend for increased NOx (Supplemental Figure 4C). In contrast, plasma ET-1 levels were increased in DIO mice compared with age-matched mice on RC diet, an outcome not observed in HF-fed *BACE1*-KO mice, which displayed values identical to RC-fed WT and *BACE1*-KO mice (Figure 5D; diet × genotype, *P* < 0.01). Additionally, increased ET-1 levels were not observed in RC-fed *hAPP_Sw_* (Figure 5D) or *hAPP23* (Supplemental Figure 4D) mice. Interestingly, ET-1 plasma levels were not altered in RC-fed WT mice infused with Aβ42 (versus ScrP controls), although short-term HF-fed mice given Aβ42 exhibited increased ET-1 compared with ScrP-infused controls (Figure 5E; diet × treatment, *P* < 0.05). The elevated ET-1 levels in DIO mice were lowered by M-3 (Figure 5F) and baseline ET-1 levels decreased in *hAPP23* mice given M-3 (Supplemental Figure 4D). These outcomes demonstrate that long-term HF feeding reduces the bioavailability of plasma NOx and raises plasma ET-1 levels. However, although elevation of plasma Aβ42 levels to that of DIO mice in RC-fed and short-term HF-fed WT mice reproduced the decrease in plasma NOx, ET-1 levels were unaffected by Aβ42 per se and appear to be upregulated primarily by HF diet, with Aβ42 playing a permissive role.

### Aortic vessel lipid deposition and structure.

Endothelial dysfunction is present in the early, preclinical, stage of atherosclerosis (13) and increased Aβ promotes atherosclerosis, inflammation, and vascular pathology (15–17). As diabetes and obesity are major risk factors for atherosclerosis, we examined whether there were indications of aortic vessel lipid deposition, inflammation, or structural alterations associated with the endothelial functional changes observed in these mouse models. Oil red O staining of aortic arches from RC- or HF-fed (5 and 12 weeks of HF diet) WT and *BACE1*-KO mice and WT mice infused with Aβ42 or ScrP for 28 days (5 weeks of HF diet) exhibited no detectable lipid deposition (Supplemental Figure 5, A–D). In addition, immunohistological analysis of perirenal aortic sections using isolectin B4 as a marker for endothelial cells and macrophages of RC- and HF-fed (12 weeks of HF diet) WT and *BACE1*-KO and WT mice infused with Aβ42 or ScrP for 28 days (5 weeks of HF diet) showed no macrophage infiltration or loss of endothelial cells (Supplemental Figure 6, A–C), with unchanged aortic wall thickness (Supplemental Figure 6D).

### Aβ42 production regulates NO signaling.

**Figure 6:**
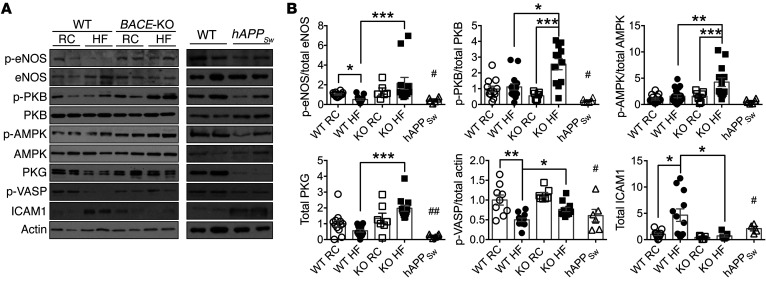
Aortic NO signaling is modified in *BACE1*-KO and *hAPP_Sw_* mice. (**A**) Representative immunoblots of p-eNOS(Ser^1177^), eNOS, p-PKB(Ser^473^), PKB, p-AMPK(Thr^172^), AMPK, PKG, p-VASP(Ser^239^), ICAM1, and actin in aortas of RC-fed WT, DIO, and RC- and HF-fed *BACE1*-KO mice (left panel), and from aortas of RC-fed WT and RC-fed *hAPP_Sw_* mice (right panel). (**B**) Ratio of signal intensities for p-eNOS to eNOS, p-PKB to PKB, p-AMPK to AMPK; and for p-VASP, PKG, and ICAM1 to actin (*n* = 6–15). Data are means ± SEM. *P < 0.05; **P < 0.01; ***P < 0.001 by Kruskal-Wallis test with Dunn’s multiple-comparisons test. ^#^*P* < 0.05, ^##^*P* < 0.01 by Mann-Whitney *U* test (vs. RC-fed WT mice).

**Figure 7:**
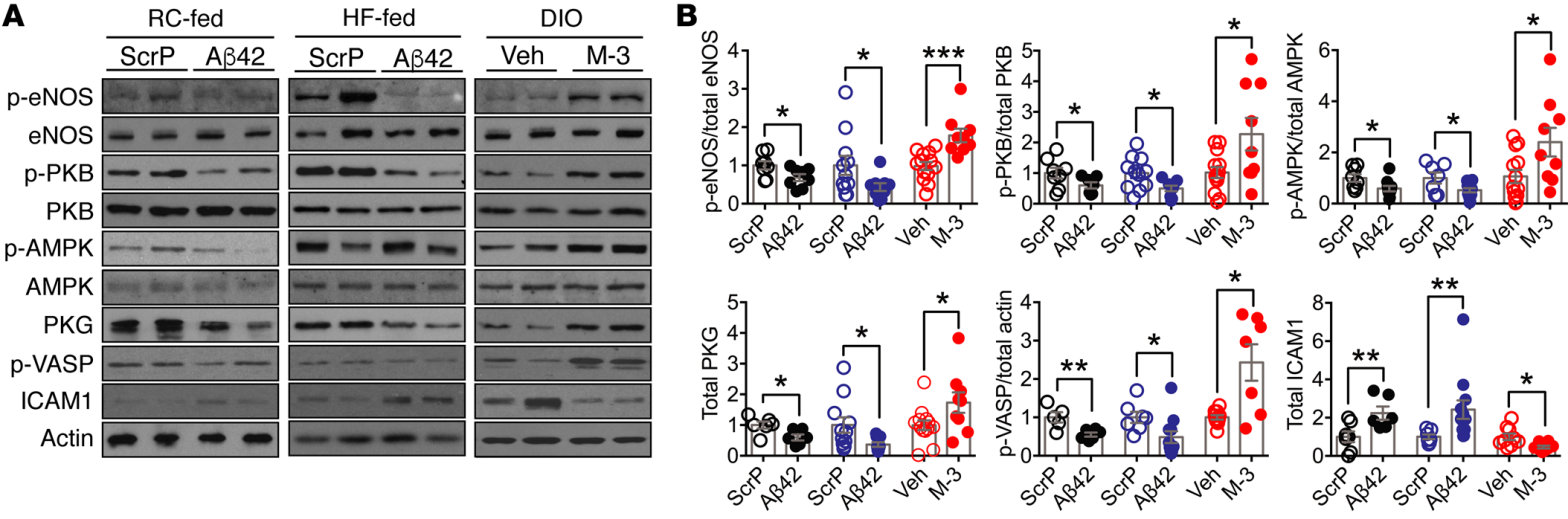
BACE1 activity and Aβ42 regulate aortic NO signaling. (**A**) Representative immunoblots of p-eNOS(Ser^1177^), eNOS, p-PKB(Ser^473^), PKB, p-AMPK(Thr^172^), AMPK, PKG, p-VASP(Ser^239^), ICAM1, and actin in aortas of ScrP- and Aβ42-treated RC-fed (left panel) and HF-fed (5 weeks) WT mice (middle panel) and in aortas of vehicle- or M-3–treated DIO mice (right panel). (**B**) Ratio of signal intensities for p-eNOS to eNOS, p-PKB to PKB, p-AMPK to AMPK; and for PKG, p-VASP, and ICAM1 to actin (*n* = 5–14). Data are means ± SEM. *P < 0.05; **P < 0.01; ***P < 0.001 by Mann-Whitney *U* test.

Next, we explored the potential mechanism(s) by which raised BACE1 activity and higher plasma Aβ42 act to regulate NO-mediated vasodilation. Vasodilation induced by NO is dependent on augmented production of cyclic GMP (cGMP) and the subsequent activation of protein kinase G (PKG) in the vascular smooth muscle, leading to relaxation. Furthermore, increased levels of plasma Aβ42 diminished vascular responses to the NO donor SNP, indicating Aβ42 alters VSMC signaling in response to NO. Consequently, we measured aortic cGMP and demonstrated that cGMP levels were lower in DIO mice (versus RC-fed controls) and in RC-fed *hAPP_Sw_* mice, but not in RC- or HF-fed *BACE1*-KO mice (Figure 5G; diet × genotype, *P* < 0.01). In addition, RC- and HF-fed WT mice infused with Aβ42 exhibited diminished aortic cGMP levels (Figure 5H; diet × treatment, *P* < 0.01), whereas DIO mice given the BACE1 inhibitor showed recovery of aortic cGMP levels (Figure 5I). In vascular endothelial cells, eNOS uses L-arginine to produce NO, which maintains blood flow and reduces inflammation. Reduced availability of L-arginine to eNOS and the resultant reduction of NO production has been implicated in the vascular dysfunction associated with diabetes, other cardiovascular disease states, and AD (33, 34). Therefore, we examined eNOS and p-eNOS (at Ser^1177^) levels, the latter as a surrogate measure of eNOS activity, in aortas by immunoblot. DIO mice displayed reduced p-eNOS levels (with no change in total eNOS) compared with RC-fed controls, whereas HF-fed *BACE1*-KO mice exhibited raised p-eNOS compared with DIO mice (Figure 6, A and B). Increased plasma Aβ42 levels, associated with *hAPP_Sw_* mice or Aβ42 infusion into short-term HF- or RC-fed mice, resulted in reduced p-eNOS levels in the aorta (Figure 6, A and B, and Figure 7, A and B). In contrast, DIO mice given a BACE1 inhibitor displayed increased aortic p-eNOS levels (Figure 7, A and B). Consequently, these data support the notion that Aβ inhibits NO production via suppression of eNOS activity.

The key enzymes involved in the phosphorylation and subsequent activation of eNOS in vascular endothelium are protein kinase B (PKB) and AMP-activated protein kinase (AMPK). Aβ has been shown to downregulate PKB expression and activity in skeletal muscle and brain, whereas suppressed AMPK activity has been implicated in AD pathogenesis (35). In general agreement with these findings, HF-fed mice with very low levels of Aβ42 (*BACE1*-KO mice) displayed enhanced p-PKB(Ser^473^) and p-AMPK(Thr^172^) levels versus DIO mice (Figure 6, A and B). Increased levels of Aβ42 were associated with reduced p-PKB in RC- or HF-fed mice and RC-fed *hAPP_Sw_* mice, with p-AMPK levels significantly reduced in RC- and HF-fed WT mice (Figure 6, A and B, and Figure 7, A and B). However, DIO mice given the BACE1 inhibitor demonstrated increased p-PKB and p-AMPK levels (Figure 7, A and B). Collectively, these results indicate that depression of Aβ42 levels in DIO mice leads to enhanced PKB and AMPK activity, increased eNOS phosphorylation, and production of NO, while increased Aβ42 tends to suppress PKB activity predominantly, correlating with reduced eNOS phosphorylation and NO levels. The enhanced levels of Aβ42 also resulted in diminished vascular responses to the NO donor SNP and depressed cGMP, indicating that Aβ42 may directly alter VSMC signaling in response to NO. Indeed, we found that total PKG levels and activity, the latter determined by monitoring changes in vasodilator-activated serum phosphoprotein (VASP) phosphorylation (Ser^239^), were reduced in mice with high Aβ42 levels (Figure 6, A and B, and Figure 7, A and B). In contrast, HF-fed *BACE1*-KO mice and DIO mice treated with M-3, with reduced levels of Aβ42, exhibited enhanced PKG and p-VASP expression levels (Figure 6, A and B, and Figure 7, A and B). In addition, intracellular adhesion molecule 1 (ICAM1), a marker of inflammation and endothelial cell activation, was shown to be increased in DIO mice (Figure 6, A and B) and in all situations (*hAPP_Sw_* mice and Aβ42-infused mice) where Aβ42 levels are increased (Figure 6, A and B, and Figure 7, A and B). In contrast, in HF-fed mice where Aβ42 is low (HF-fed *BACE1*-KO mice and DIO mice treated with M-3), ICAM1 levels were significantly decreased (Figure 6, A and B, and Figure 7, A and B).

### Blood pressure.

We also determined whether the impairments in vascular reactivity induced by increased plasma Aβ42 also result in changes in blood pressure (BP) in mice. Although BP studies on conscious mice are preferred, mice with infusion pumps were anesthetized for this measurement, resulting in depressed arterial pressure (36). *BACE1*-KO mice are normotensive on an RC diet; however, loss of BACE1 protects the mice against 20-week HF diet-induced hypertension (Table 1), as well as maintaining a leaner body weight and lower fasted blood glucose (Table 1 and ref. 26). Furthermore, treatment of DIO mice with a BACE1 inhibitor also reduced systolic and diastolic BP (Table 2) and lowered fasted blood glucose and body weight, as previously reported (30). In contrast, *hAPP_Sw_* mice on RC diet displayed raised systolic and diastolic BP (Table 1) and direct infusion of Aβ42 also resulted in increased BP in anesthetized RC- and HF-fed mice versus ScrP controls (Table 2). In general, these manipulations had no effect on heart rate, apart from *hAPP_Sw_* mice, which displayed a significantly increased heart rate and the previously recognized chronotropic effect of isoflurane (37). Taken together, these data show that the adverse effects induced by Aβ42 on the vasculature also result in increased systemic BP (but not heart rate), indicating the potential use for BACE1 inhibitors to treat obesity- and diabetes-induced hypertension.

### Plasma Aβ42 and human endothelial function.

The T2D (*n* = 220) and control (*n* = 127) groups (Human Study 2) were matched for age (65.1 ± 8.2 and 66.0 ± 8.3 years, respectively), mean arterial BP (95.6 ± 10.8 and 97.0 ± 12.2 mmHg, respectively), and sex (male/female = 142:85 and 75:53, respectively). BMI was significantly greater in patients with T2D (32.5 ± 5.67 and 28.4 ± 4.3, respectively, *P* = 0.001). Duration of diabetes was 11.2 ± 7.4 years. Plasma Aβ42 levels were significantly greater in patients with T2D compared with values in the control group (12.9 ± 4.3 and 10.8 ± 3.9 pg/mL, *P* < 0.001). Endothelial function (reactive hyperemia index, RHI) showed significant univariate associations with HbA1c (*r* = –0.271, *P* < 0.0001), BMI (*r* = –0.259, *P* < 0.0001), female sex (*r* = 0.159, *P* = 0.003), Aβ42 (*r* = –0.142, *P* = 0.008), age (*r* = 0.135, *P* = 0.011), and systolic BP (*r* = 0.132, *P* = 0.013). In a stepwise linear regression model, entering HbA1c, BMI, sex, Aβ42, age, and systolic BP, endothelial function showed an independent association with BMI (β = –0.215, *P* < 0.001), female sex (β = 0.211, *P* < 0.0001), systolic BP (β = 0.149, *P* = 0.003), HbA1c (β = –0.183, *P* = 0.001), and Aβ42 (β = –0.113, *P* = 0.028), indicating the findings in mice may also be relevant to human diabetes and vascular disease.

## Discussion

Various reports indicate that exogenously applied soluble Aβ40 and/or Aβ42 mediate vasoactive effects, through inducing vasoconstriction and/or attenuating endothelium-dependent vasodilation (15, 38). Furthermore, in vitro studies suggest that the latter outcome may be due, in part, to the inhibition of NO production through decreased levels of p-eNOS at Ser^1177^ in endothelial cells (19, 20, 39). However, the translation of these in vitro findings to the whole-body animal and human situation is questionable given that high (~μM) concentrations of Aβ peptides were applied in many of these studies, orders of magnitude above that reported in tissue and blood samples from humans and animals under physiological or pathophysiological conditions. In the present study, we demonstrate that chronic HF feeding of mice, causing obesity and hyperglycemia, raises BACE1 activity as monitored by changes in sAPPβ levels, although the levels of this peptide may also be affected by rates of degradation or recycling through endosomes, which were not assessed. Nevertheless, we show that HF feeding increases Aβ42 peptide levels, which do not exceed approximately 2–10 pM in plasma, with no change in Aβ40 levels. A similar magnitude of increase in plasma Aβ42, and not Aβ40, was also observed in a small cohort of patients with diabetes and displayed a positive correlation with the level of hyperglycemia. The DIO mice in our study exhibited markedly reduced vascular responsiveness to ACh, which was mirrored in RC-fed mice by raising the circulating level of Aβ42 to that observed in plasma of HF-fed mice, directly by Aβ42 infusion or indirectly through increased BACE1-dependent cleavage of human mutant APP in transgenic mouse models. Importantly, this effect of increased Aβ42 was independent of HF feeding, obesity, and hyperglycemia. In contrast, in mice lacking BACE1 with very low levels of Aβ42 there was no loss of ACh vasodilator responsiveness. Furthermore, in DIO mice chronically treated with a BACE1 inhibitor to lower Aβ42 levels, there was almost complete restoration of the ACh vasodilator responsiveness accompanied by reduced BP. These data strongly support the notion that increased Aβ42 levels result in vascular dysfunction, leading to more constricted blood vessels and contributing to a hypertensive state. In addition, the aortic vessel walls from HF-fed or Aβ42-infused mice demonstrated no lipid deposition, similar thickness with uniform layers of smooth muscle and intact endothelial cell layer, and no significant inflammatory infiltrate. Consequently, impairment of endothelium-dependent vasodilation by elevated plasma Aβ42 is measurable before any histological abnormalities are detected in the vessel wall.

Our in vivo mouse study demonstrates that increased levels of Aβ42 result in decreased NO production, which appears not to be regulated by a change in total eNOS, but by modulation of eNOS activity through a reduction in phosphorylation at Ser^1177^. This is in line with previous work showing that acute application of Aβ reduces NO release, inhibits eNOS activity, and prevents ACh-induced phosphorylation of eNOS, at Ser^1177^, in cerebral vessels (20, 39). The posttranslational modification of eNOS at this site may be mediated by PKB and/or AMPK activity, although the finding that increased Aβ42 was associated with diminished PKB (as detected through p-PKB levels), rather than AMPK (p-AMPK levels), activity indicates this pathway as the predominating site of action. This is supported by a previous in vitro study using cultured endothelial cells, which showed that picomolar levels of soluble Aβ inhibits PKB phosphorylation and p-eNOS (37), which may occur by Aβ interfering with the formation of the activation complex of PKB with phosphoinositide-dependent kinase-1 (PDK-1) (40).

In contrast to the apparently direct effects of Aβ42 on NO production, production of ET-1 is not increased by Aβ42 under RC-fed conditions in mice by direct infusion of WT mice with mouse Aβ42 or in AD mouse models overexpressing mutant human APP, but is increased by HF diet challenge, an action exacerbated by the presence of Aβ42. This outcome suggests that the vascular dysfunction observed in RC-fed mice associated with increased Aβ42 and in AD mouse models is primarily driven by NO depletion. ET-1 levels and activity are enhanced, in association with increased vasoconstrictor tone, in people with obesity, T2D, and the metabolic syndrome (41). Moreover, hyperglycemia enhances ET-1 expression and release in human and rodent endothelial cells (42, 43). Under physiological conditions ET-1 production is tightly controlled, whereby increased production of NO inhibits expression and production of ET-1 (44). Thus, increased levels of Aβ42, caused by chronic HF feeding and hyperglycemia or by excessive BACE1 cleavage of mutant APP, as in many familial AD cases and numerous AD rodent models (e.g., *hAPP_Sw_* and *hAPP23*), will directly diminish NO bioavailability and indirectly elevate ET-1 levels. Consequently, reducing plasma and tissue Aβ levels should have a substantial beneficial effect on obesity- and diabetes-impaired vasoreactivity. In agreement with this notion, we show that treating DIO mice with a BACE1 inhibitor reduced circulating levels of Aβ42, restored vascular reactivity, and decreased BP contemporaneously with elevation of NO levels and suppression of ET-1 production.

Therefore, our results strongly suggest that excessive Aβ induces endothelial dysfunction. However, impaired vasodilator responses in mice with high levels of Aβ42 (i.e., DIO, RC-fed *hAPP_Sw_*, *hAPP23*, and Aβ42-infused mice) to the NO donor SNP also indicates endothelium-independent actions of Aβ42. NO induces vascular relaxation by stimulating production of cGMP by soluble guanylate cyclase (sGC), resulting in PKG activation and VSMC relaxation. The findings that cGMP, PKG, and p-VASP levels in mouse aortas are decreased in mice with raised levels of Aβ42 and recovered in DIO mice treated with the BACE1 inhibitor support such a direct link. Indeed, it has been reported that Aβ can downregulate the expression of sGC in astrocytes, rendering them less sensitive to SNP (45) and that high (μM) Aβ suppresses NO-stimulated sGC activity (and lowers cGMP) in porcine smooth muscle cells and human Jurkat cells (46). Thus, HF feeding and hyperglycemia with subsequent elevated BACE1 activity and Aβ42 levels inhibits vasodilation and promotes vasoconstriction by multiple mechanisms, leading to a hypertensive state. In addition, individuals with AD have reduced plasma NO levels and elevated asymmetric dimethylarginine (ADMA), a natural inhibitor of NO production (47).

Various studies have demonstrated that Aβ40 and/or Aβ42 induces vascular dysfunction and there is a lack of clarity as to the species responsible for direct vascular effects. In this study, we find that Aβ42, but not Aβ40, levels were enhanced by chronic HF feeding, which correlates well with endothelial dysfunction, suggesting that Aβ42 may be responsible. It is well documented that the aggregation state or “aging” of Aβ peptides has a major influence on their biological effects. However, most preceding studies applied high concentrations of freshly made monomeric human Aβ peptides to tissues or cells, thus lessening the opportunity for formation of aggregated states. In the present study, we infused monomeric murine Aβ42 (which is less aggregation prone than human Aβ42) and have not made any assessment of endogenous or exogenous Aβ aggregation status, although it is likely that, over a 4-week exposure period at 37°C in plasma and tissues, a significant proportion of the infused murine Aβ42 will no longer be in the monomeric state. Importantly, mice overexpressing human mutant APP isoforms from birth, on an RC diet, exhibit similar changes in endothelium-dependent and -independent vasodilation, indicating that sufficient Aβ42 is present in either scenario to cause vascular dysfunction, regardless of aggregation status. The source of endogenous plasma and peripheral tissue Aβ42 is uncertain. BACE1 expression and activity is highest in brain and Aβ peptides are transported through the blood-brain barrier (BBB) to the plasma, an action potentially exacerbated by BBB damage associated with cerebrovascular pathologies. However, it is increasingly recognized that BACE1, APP, and γ-secretase proteins are also expressed in many peripheral tissues, with blood platelets having the highest APP expression after brain and are a significant source of plasma Aβ (48). In addition, endothelial cells and VSMCs express APP and various secretases responsible for cleavage and may therefore contribute directly to tissue and plasma Aβ load under physiological and pathophysiological conditions (49, 50).

Our findings in mice of an important role for BACE1 and Aβ42 in vascular dysfunction can be translated to humans, where we show localization of BACE1 protein in endothelial cells and VSMCs in human temporal artery, with increased BACE1 transcript levels in internal mammary arteries associated with obesity, but not diabetes. We also find an association between elevated plasma Aβ42 in patients with T2D and a positive correlation with glycated hemoglobin, suggesting Aβ42 may be a contributing factor or marker of hyperglycemia. Furthermore, raised plasma Aβ42 correlates negatively with endothelial function in humans. Thus, the obese state may increase BACE1 transcription, whereas diabetes (hyperglycemia) and obesity associate with raised BACE1 activity and higher levels of plasma and tissue Aβ42 in humans and mice (30). These findings open up avenues for further exploration of the possible role of amyloid proteins in development of vascular dysfunction in obesity and T2D and the potential of examining BACE1 inhibitor drugs on vascular dysfunction.

In conclusion, we have demonstrated that metabolic disorder in mice and humans, associated with obesity and diabetes, results in elevated plasma Aβ42, which is correlated with the presence of peripheral endothelial and vascular dysfunction. This increased plasma Aβ42 results in diminished NO production and increased ET-1, leading to depression of endothelium-dependent vasorelaxation and increased vasoconstriction, which in turn drives local tissue ischemia and further increased BACE1 activity and Aβ42 levels, creating a vicious cycle associated with progressive vascular dysfunction. Future studies are required to determine whether human plasma Aβ peptides may be useful measures in a panel of biomarkers for early diagnosis of vascular disease more generally, both in the brain (e.g., dementia) and periphery (e.g., atherosclerosis, hypertension). Additionally, repurposing BACE1 inhibitors currently undergoing clinical trials for AD for moderation of peripheral Aβ levels could be a novel therapy for vascular dysfunction induced by metabolic disease.

## Methods

### Human study populations.

Study 1: The Genetics of Diabetes Audit and Research in Tayside Scotland (GoDARTS) project was established in 2005 to identify genetic risk factors for diabetes and its complications (51). Participants, including individuals with and without diabetes, are all required to complete a lifestyle questionnaire, a baseline clinical examination, and provide their biological samples. All participants provided broad informed consent for their health information from the National Health Service (NHS) and biological samples to be anonymously linked to the study for future scientific research. The linked health information includes their personal health status, their general practice clinic visits, outpatient appointments, prescribing history, and hospital admissions.

Study 2: All participants gave informed written consent. Participants in this study were recruited as part of the “surrogate markers for micro- and macro-vascular hard endpoints for innovative diabetes tools” (SUMMIT) study (IMI grant number 115006; http://www.imi-summit.eu) from the Scottish Primary Care Research Network, the Scottish Diabetes Research Network, Secondary Care Diabetes Clinics, and through advertising such as posters and leaflets. Details of the SUMMIT study and plasma sampling are described in detail elsewhere (52).

Two groups of participants were included: (a) Patients with T2D with or without diagnosed cardiovascular disease. (b) A control group of subjects without T2D but matched for gender and age (±5 years) and type of cardiovascular disease complication.

Presence of cardiovascular disease included a medical history of myocardial infarction, percutaneous coronary intervention, coronary arterial bypass graft, unstable angina, and specialist-diagnosed cerebrovascular event from Ninewells Hospital, Dundee.

### Assessment of endothelial function in humans.

Endothelial function was measured using an EndoPat (Itamar Medical), as described previously (53). In brief, the index fingers or middle fingers were placed in pneumo-electric tubes and arterial pulsatile volume changes were recorded continuously from both hands. Following 8 minutes of rest, a BP cuff on the upper arm was inflated to 200 mmHg for 5 minutes to induce ischemia. On release of the pressure cuff, the subsequent reactive hyperemia and arterial dilation mediated was recorded for 10 minutes. The reactive hyperemia index (RHI) was calculated and expressed as a ratio of the postocclusion to preocclusion signal amplitudes.

### Assessment of human mammary artery transcripts.

Human studies included 115 prospectively enrolled patients undergoing coronary artery bypass graft (CABG) surgery at the John Radcliffe hospital, Oxford University NHS Foundation Trust, United Kingdom. Exclusion criteria included any active inflammatory, neoplastic, renal, or hepatic disease. During surgery, segments of internal mammary artery (IMA) were collected, transferred to the lab on ice, and snap-frozen at –80°C for subsequent gene expression studies. All patients provided written informed consent before enrolment. The demographic characteristics of the participants are presented in Supplemental Table 1. IMA samples were harvested at the time of CABG and transferred to the lab in oxygenated (95% O_2_/5% CO_2_) ice-cold Krebs-Henseleit buffer. There, the vessel lumen was flushed gently with an insulin syringe to remove excess blood. Vessels were then separated from the surrounding adventitial and adipose tissue under magnification. The same anesthetics were used in all cases, and each sample was always obtained at the same stage of the operation, to limit interpatient variability. IMA samples were finally snap-frozen in TRI reagent (MilliporeSigma) and stored at –80°C until used for RNA isolation. Total RNA was isolated by phenol/chloroform (1:5 ratio) separation followed by isopropanol precipitation and 70% ethanol cleanup. RNA concentration and quality were evaluated spectrophotometrically on a NanoDrop ND-1000. RNA was reverse transcribed to cDNA by using SuperScript VILO Master Mix (Thermo Fisher Scientific) following the manufacturer’s instructions and extending the cDNA synthesis step to 2 hours at 60°C on a Veriti thermal cycler (ABI). Quantitative real-time PCR was performed by TaqMan chemistry, using the standard universal TaqMan protocol as indicated by the manufacturer, on a QuantStudio 7 flex real-time PCR system (Thermo Fisher Scientific). All samples were run in duplicate using 5 ng of cDNA as starting mass. A number of standard dilutions of a pooled vascular cDNA sample were run on the same plate as the experimental samples in order to calculate the efficiency of the probes used; data analysis was then performed by the Pfaffl method. *GAPDH* was used as housekeeping gene for human vascular tissue. The IDs of the TaqMan probes used are: *GAPDH*, Hs02786624_g1; *BACE1*, Hs01121195_m1.

### Mouse studies.

Mice were given free access to food and water and maintained on a 12-hour light/dark cycle. Male hemizygotic B6.129-Tg(APPSw)40Btla/Mmjax (JAX 34831; transgenic [Tg] mice expressing human APP containing the familial AD Swedish double mutation K670N/M671L; *hAPP_Sw_*) or male hemizygotic B6.Cg-Tg(Thy1-APP)3Somm/J (JAX 030504; Tg mice expressing human APP with the Swedish double mutation under the Thy1 promoter; *hAPP23*) were obtained from the Jackson Laboratory (29, 32). Male and female *BACE1*-Tg transgenic mice on the C57BL/6J background (backcrossed >10 times) provided *BACE1*-KO mice and WT control littermates (26). For dietary studies, mice were fed RC (4% calories from fat: RMI 505) throughout the duration of study or at 8–10 weeks old were switched to a HF (45% calories from fat: SDS 824053) diet (see Supplemental Table 2 for further details of composition) for the remainder of the study (i.e., 5 weeks or 20 weeks [referred to as DIO HF feeding]). Only males were used in the APP, M-3, and Aβ studies. However, both male and female mice were used in the *BACE1*-KO studies, with roughly equal percentages of each sex in each group. Osmotic minipumps (2004 model, Alzet) were implanted under inhaled isoflurane anesthesia (1 L/min) and delivered the BACE1 inhibitor M-3 (10 mg/kg/day) (54) or vehicle (50:50 DMSO/phosphate-buffered saline [PBS]) subcutaneously (s.c.) for 28 days into WT mice fed an HF diet for 20 weeks (DIO mice). Aβ42 or scrambled peptide (ScrP; 3.36 μg/kg) was infused s.c. for 28 days via an osmotic minipump into 12-week-old WT mice on RC diet or WT mice fed an HF diet for 1 week before surgery. These latter mice, along with *hAPP_Sw_* and *hAPP23* mouse groups, had their microvascular function determined at approximately 16 weeks of age.

### Assessment of microvascular function in mice.

Two days before the measurements, hair was removed from the flank area of the mouse using a combination of shaving and commercially available depilatory cream (Nair). For microvascular assessments, mice were anesthetized (isoflurane, Abbott Laboratories) and body temperature was maintained at 37°C using a heat mat before iontophoresis of phenylephrine (PE), ACh, or SNP. A laser Doppler imager (LDI, Moor Instruments) was used to measure skin microvascular perfusion noninvasively. The LDI performs scans continuously during the iontophoresis period and provides a measure of microvascular blood perfusion (in arbitrary perfusion units [AU]) as a function of the number of red blood cells multiplied by their mean velocity. Color-coded images are generated for each perfusion scan and numerical outputs are produced using proprietary software (moorLDI software, version 5.3, Moor Instruments). An iontophoresis chamber (ION6 probe, Moor Instruments), consisting of a 20-mm internal diameter ring with a wire electrode running around the inner surface, was attached to the flank using double-sided adhesive tape. A reference electrode pad was attached to the underside of the animal to complete the iontophoresis circuit. To standardize baseline perfusion, blood vessels were preconstricted with iontophoresis of 1% PE for 5 minutes (current = 100 μA), followed by iontophoresis of 2% ACh (endothelium-dependent) for 10 minutes (current = 100 μA). At a different site on the opposite flank, 2% SNP (endothelium-independent) was delivered by iontophoresis for 10 minutes (current = 100 μA). To determine the contribution of endothelium-derived NO to ACh-mediated vasodilation, microvascular responses to ACh were assessed after pretreatment with the nonselective inhibitor of NO synthase, L-NAME (MilliporeSigma). L-NAME (20 mg/kg) was administered i.p. 30 minutes before assessment of ACh-mediated vasodilation (52).

To determine the maximum microvascular dilator capacity, a hyperemic response was initiated, on a separate occasion, by localized heating of the skin to 42°C using a specially designed heating probe (SH02 Skin Heating Unit and SHP3 probe, Moor Instruments). Animals were anesthetized as described above and a skin heating probe with an internal diameter of 20 mm and an inner ring heating electrode providing a total surface area of 3.2 cm^2^ was attached to the flank using double-sided adhesive rings (IAD, Moor Instruments). Baseline measurements of perfusion were taken for 5 minutes using the LDI, followed by continual assessment of microvascular responses to localized heating of the skin as the temperature within the heating chamber was increased at a rate of 1°C/min until a maximum temperature of 44°C was achieved. This was maintained for a period of more than 10 minutes, which was sufficient to induce a plateaued maximum vasodilation in the skin microcirculation. Perfusion images were analyzed using propriety software (Moor Instruments, version 5.3) and are expressed as percentage delta response, which was calculated as (maximum vasodilation in response to ACh [or SNP] – maximum vasoconstriction in response to PE [i.e., a baseline arbitrarily set to 100%]). For localized heating, data are expressed as percentage change from baseline perfusion at 25°C–30°C.

### BP.

BP and heart rate were assessed at the terminal time point using the CODA noninvasive blood pressure system (Kent Scientific). This system consists of an occlusion cuff and a volume pressure-recording (VPR) cuff placed around the tail of the animal. Mice that underwent minipump implantation had BP measurements made under anesthesia.

### Protein expression and BACE1 activity.

Aortas were dissected, cleaned, washed in ice-cold PBS, and snap-frozen in liquid nitrogen. Tissue was homogenized using a probe sonicator (Soniprep 150, MSE) in lysis buffer (26). For immunoblotting, 20 μg aortic lysate was separated by SDS-PAGE and transferred to nitrocellulose membranes. The details of all primary antibodies used for immunohistochemistry, immunofluorescence, or immunoblot, suppliers, species, and dilution are shown in Supplemental Table 3. BACE1 activity was determined, indirectly, by measuring plasma and tissue levels of the shed soluble product of APP cleavage, sAPPβ.

### Immunohistochemistry.

Thoracic aorta was dissected and cleaned of perivascular fat before fixation in neutral buffered 10% formalin for 48 hours. Tissues were embedded in paraffin before 4-μm-thickness sectioning produced using a microtome (Leica). Formalin-fixed, paraffin-embedded human temporal artery sections were provided by Tayside Biorepository from the Tayside pathology archive. Mouse and human sections were stained for BACE1 (1:3000, N1C1, GeneTex). For immunofluorescence studies, mice were perfused with PBS followed by formalin. Aortas were dissected and cleaned of adventitial tissue and stored in 10% formal saline. Aortas were paraffin embedded and sectioned at 14 μm. Sections were baked at 60°C for 2 hours, before dewaxing. Antigen retrieval was performed using trisodium citrate buffer, pH 6, and placed in pressure cooker for antigen retrieval. Sections were permeabilized in PBS plus 0.5% Triton X-100 for 10 minutes, and then blocked for 1 hour (PBS + 0.2% BSA+ 10% normal goat serum). Following a PBS wash, sections were incubated with primary antibodies (BACE1, smooth muscle actin [SMA], or CD31) diluted in PBS plus 0.2% BSA for 1 hour at room temperature. Following removal of primary antibodies, sections were washed 3 times, 5 minutes each in PBS before incubation with secondary antibodies (Alexa Fluor 488 goat anti-mouse, Alexa Fluor 594 goat anti-rabbit, 1:500; Thermo Fisher Scientific) plus 1 μg/mL Hoechst for 1 hour at room temperature. Following removal of secondary antibodies, sections were washed 3 times, 5 minutes each in PBS before mounting with Vectashield H1000, coverslips sealed with nail polish, and stored at 4°C until imaged.

### Analysis of aortic structure.

Mice were terminally anesthetized and perfusion-fixed with 4% paraformaldehyde (PFA) in PBS (pH 7.2) (Thermo Fisher Scientific). Entire aortic trees were harvested and stored for up to 7 days in 4% PFA solution to ensure complete fixation. The aortic arch was removed and dissected free of fat and other tissue and stained with Oil Red O (MilliporeSigma). Stained aortas were twice washed in 60% isopropanol, opened, and mounted en face before being imaged digitally using an Olympus SZ61 dissecting microscope (magnification: ×12; Olympus Corporation) and QImaging QICAM Fast 1394 camera (Teledyne QImaging). Lipid burden was quantified from these images using Image-Pro Plus 6.0 software (Media Cybernetics). The perirenal section of the aorta was embedded in Optimal Cutting Temperature compound (VWR International) and 10-μm sections produced using a cryostat (Leica Biosystems). Sections were stained for VSMCs using a rabbit anti–α-smooth muscle actin primary antibody and a goat anti-rabbit Alexa Fluor 647 secondary antibody (A21246, 1:200; Thermo Fisher Scientific). Sections were costained with isolectin-B4 conjugated to Alexa Fluor 488 (I21411, 1:100; Thermo Fisher Scientific) as an endothelial and macrophage marker. Slides were then mounted using DAPI Fluoromount-G mounting medium (0100-20; Southern Biotech). Imaging was performed using a Zeiss LSM880 confocal microscope with ZEN Black Software (Zeiss) and images analyzed using ImageJ open-source software (NIH).

### Aβ and sAPPβ measurement.

Aβ40, Aβ42, and sAPPβ were measured in mouse plasma and aortic lysate by ELISA as per the manufacturer’s instructions (Meso Scale Discovery). Human plasma Aβ40 and Aβ42 in T2D patients and controls (Study 1: GoDARTS samples) were measured by ELISA (WAKO), with the operator blinded to group membership, performed before obtaining the Simoa platform. The Aβ42 samples were measured after an 8-fold dilution with an interassay variation of 1.49% and a lower limit of quantification (LLOQ) of 0.1 pg/mL. Human plasma Aβ42 (Study 2: SUMMIT samples) was determined in duplicate from each sample using the Simoa HD-1 analyzer (Quanterix) by the University of Dundee immunoassay core facility staff, who were blinded to group membership, using a human Aβ42 assay kit (Simoa Human Aβ42). The Aβ42 samples were measured after a 4-fold dilution with an interassay variation of 5.4% and a LLOQ of 0.137 pg/mL.

### Blood chemistry.

Plasma total NO (NOx) concentration was determined using a total NO assay kit (Enzo Life Sciences). Plasma samples underwent ultrafiltration through a 10,000 MWCO filter (Merck Millipore) before quantification. ET-1 levels were measured by ELISA (R&D Systems). cGMP levels were measured in aortic lysates by ELISA (Cayman Chemical) following purification with 6% trichloroacetic acid and acetylation using 4 M potassium hydroxide. Blood glucose was measured in overnight-fasted mice using a glucometer (Bayer).

### Statistics.

Data sets were analyzed for statistical significance using GraphPad Prism 6. Sample size was determined according to our previous studies and published reports describing similar experimental procedures. For mouse studies, the investigators were not blinded to treatment groups. All vascular studies and subsequent analyses on patients were carried out by operators who were blinded to the status of the individual with respect to presence of diabetes or cardiovascular disease. Values are shown as means ± SEM, except for results from the SUMMIT study, which are presented as means ± SD. Significance (*P* ≤ 0.05) was determined by unpaired Student’s *t* test, when 2 groups were compared. When 3 or more groups were compared 2-way ANOVA (Tukey’s multiple-comparisons post hoc test) examined the main factors of diet and genotype for an interaction. All data were tested for normality using the Shapiro-Wilk normality test. All data, apart from densitometry analysis of immunoblots, passed this test (*P* > 0.05). Consequently, the immunoblot data were analyzed for 2 or multiple groups using Mann-Whitney *U* and Kruskal-Wallis tests, respectively. All normally distributed data sets containing *n* > 6 were tested for outliers using the Grubbs test. The association between endothelial function in the human study and Aβ42 and other parameters was assessed using linear regression.

### Study approval.

All animal care, experimental protocols, and procedures were performed in accordance with the Animal Scientific Procedures Act (1986), with approval of University of Dundee Ethics Committee. For human studies 1 and 2, research conformed to the declaration of Helsinki and was approved by Tayside Committee on Medical Research Ethics (REC reference 053/04) and the East of Scotland Research Ethics Service (Tayside Committee; 10/S1402/44), respectively. For IMA samples, the study protocols agreed with the Declaration of Helsinki principles and were approved by the local Research Ethics Committee (Oxford REC 11/SC/0140). Ethical approval for human temporal artery samples was obtained through Tayside Biorepository (TR382).

## Author contributions

PJM, BMC, ZT, CM, IA, JB, FP, CHO, NYY, NM, AS, AP, ADM, and MS performed the experiments. MLJA, PJM, FK, CA, KJG, and CNAP designed the studies. All authors analyzed and commented on the data. MLJA and PJM wrote the manuscript with input from all authors.

## Supplementary Material

Supplemental data

## Figures and Tables

**Table 2:**
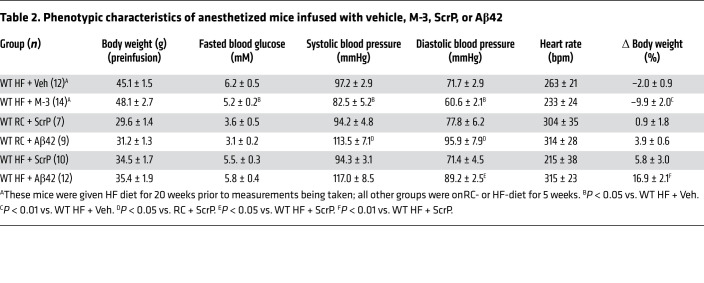
Phenotypic characteristics of anesthetized mice infused with vehicle, M-3, ScrP, or Aβ42

**Table 1:**
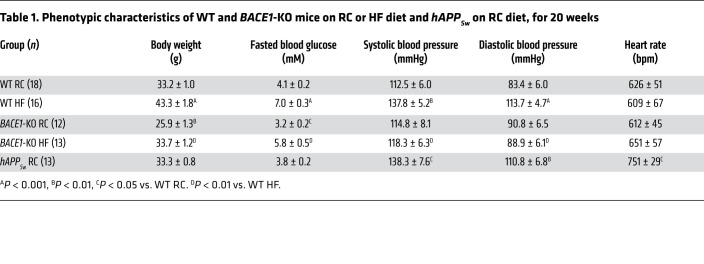
Phenotypic characteristics of WT and *BACE1*-KO mice on RC or HF diet and *hAPP_Sw_* on RC diet, for 20 weeks

## Version 1 05/14/2020

In-Press Preview

## Version 2 06/29/2020

Electronic publication

## Version 3 07/21/2020

CCBY information corrected in copyright

## Version 4 08/03/2020

Print issue publication
