# Expression of Concern for Elevated circulating amyloid concentrations in obesity and diabetes promote vascular dysfunction

**DOI:** 10.1172/JCI199125

**Published:** 2025-09-02

**Authors:** Paul J. Meakin, Bethany M. Coull, Zofia Tuharska, Christopher McCaffery, Ioannis Akoumianakis, Charalambos Antoniades, Jane Brown, Kathryn J. Griffin, Fiona Platt, Claire H. Ozber, Nadira Y. Yuldasheva, Natallia Makava, Anna Skromna, Alan Prescott, Alison D. McNeilly, Moneeza Siddiqui, Colin N.A. Palmer, Faisel Khan, Michael L.J. Ashford

Original citation: *J Clin Invest*. 2020;130(8):4104–4117. https://doi.org/10.1172/JCI122237

Citation for this Expression of Concern: *J Clin Invest*. 2025;135(17):e199125. https://doi.org/10.1172/JCI199125

The Editors recently became aware of blot anomalies in Figures 6A and 7A. The Editors have requested an institutional investigation into this matter, and we will inform our readers of the outcome when the investigation is complete.

## Version 1 09/02/2025

Electronic publication

