# Supplementary material for: Elevated circulating amyloid concentrations in obesity and diabetes promote vascular dysfunction
Source: J Clin Invest. 2020 Jun 29;130(8):4104–17. doi: 10.1172/JCI122237 (PMC7410081; doi:10.1172/JCI122237)
Supplement: Supplemental data [file jci-130-122237-s229.pdf]

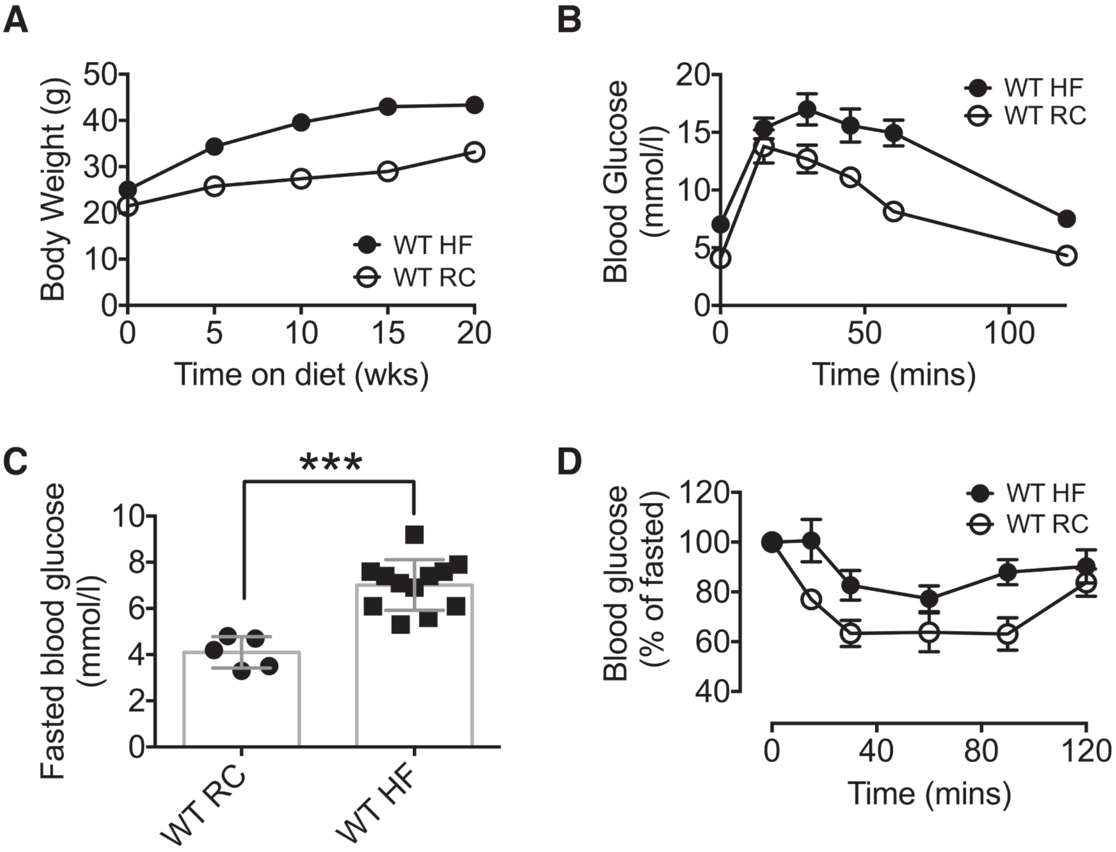

Supplemental Figure 1. High fat fed WT mice exhibit increased body weight, impaired glucose disposal higher fasted glucose and reduced insulin sensitivity.

(**A**) Body weight curves of age-matched (8-10 weeks old) WT littermate mice following 20 weeks of regular chow (RC: *n*=11) or high fat (HF: *n*=15) diet . (**B**) Intraperitoneal glucose tolerance on RC-fed (*n*=5) and HF-fed (*n*=12) WT mice performed on week 19 of dietary intervention. (**C**) Fasted blood glucose levels of WT mice on RC or HF diet for 19 weeks. (**D**) Intraperitoneal insulin tolerance on RC-fed (*n*=8) and HF-fed (*n*=6) WT mice performed on week 20 of dietary intervention. Data are means ± SEM. ***$P < 0.001$ by 2-tailed unpaired Student's t-test.

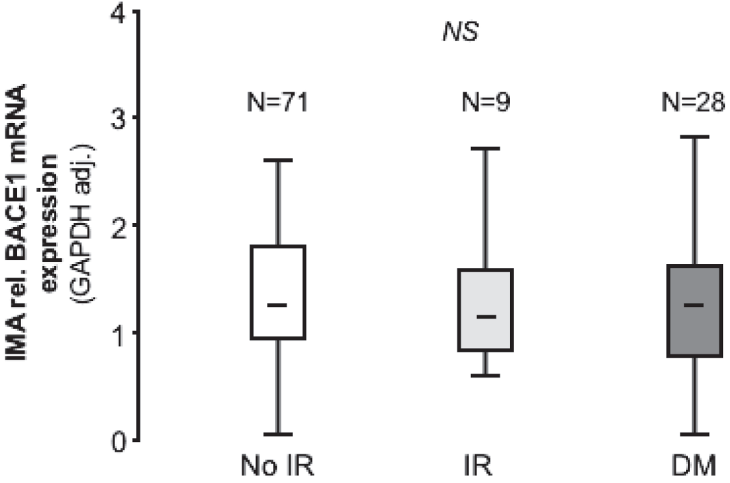

Supplemental Figure 2. Internal mammary artery BACE1 mRNA expression as a function of diabetes and insulin resistance.
IR: insulin resistance (defined by HOMA-IR > 2.8). DM: diabetes mellitus.
NS: non-significant. Data presented as means ± SD.

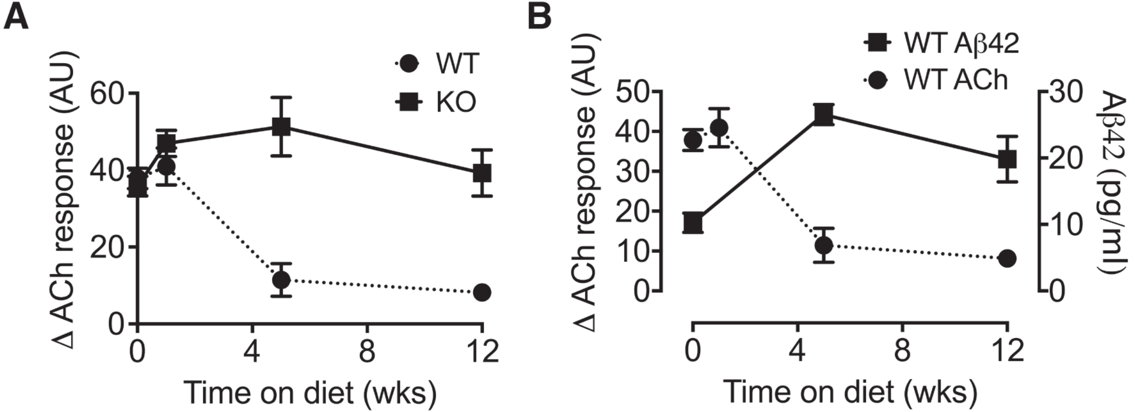

Supplemental Figure 3. ACh response size and plasma Aβ_{42} with time on HF diet. (**A**) Endothelium-dependent microvascular responses induced by ACh in HF-fed WT and *BACE1*^{KO} mice as a function of time on diet (*n*=6-18). (**B**) Endothelium-dependent microvascular responses induced by ACh with corresponding plasma Aβ_{42} levels in HF-fed untreated WT mice as a function of time on diet (*n*=9-19). Data are means ± SEM.

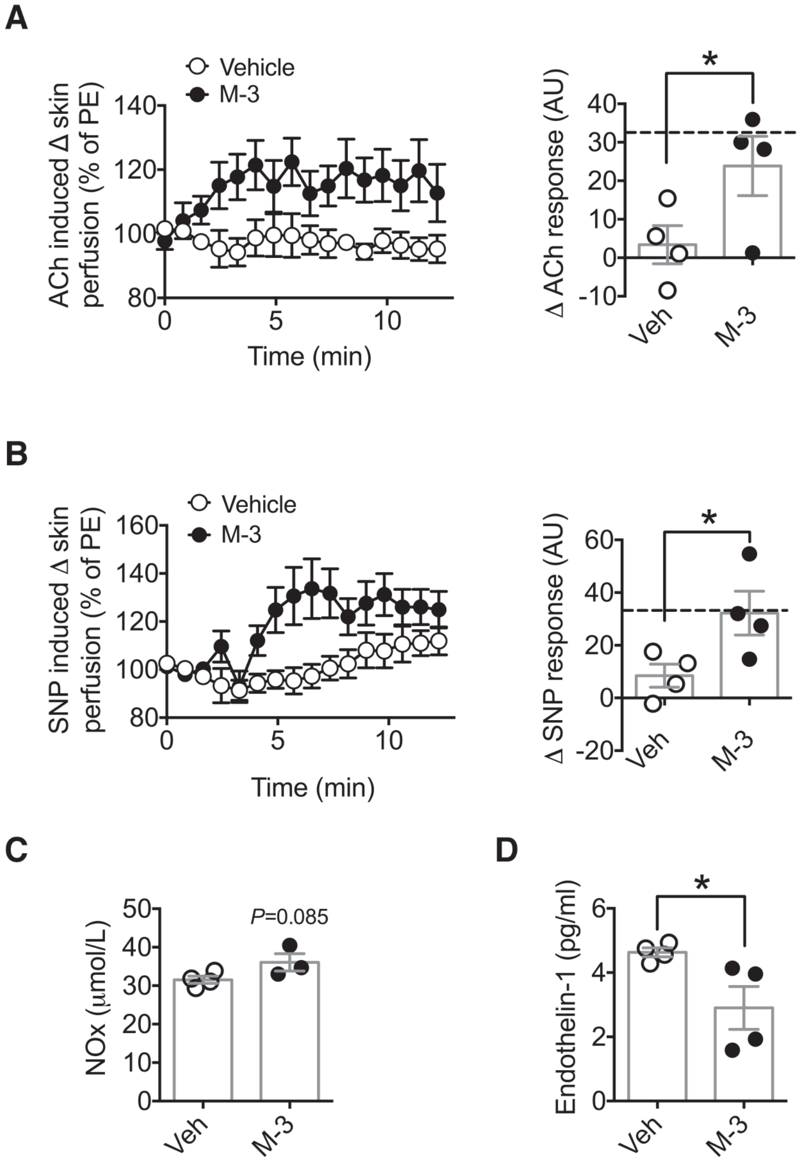

Supplemental Figure 4. M-3 improves microvascular function in *hAPP23* mice. (**A**) Endothelium-dependent microvascular responses induced by ACh in RC-fed *hAPP23* mice (*n*=4) after 4 weeks infusion of vehicle or M-3, with quantitative analysis of ACh response. The broken line denotes the RC-fed *BACE1*^{KO} ACh response. (**B**) Endothelium-independent responses induced by SNP in RC-fed *hAPP23* mice (*n*=4) after 4 weeks infusion of vehicle or M-3, with quantitative analysis of SNP response. The broken line denotes the RC-fed *BACE1*^{KO} SNP response. (**C**) Plasma NO levels in vehicle and M-3 treated *hAPP23* mice (*n*=3-4). (**D**) Plasma ET-1 levels in vehicle and M-3 treated *hAPP23* mice (*n*=4). Data are means ± SEM. * $P < 0.05$ by 1-tailed unpaired Student's t-test.

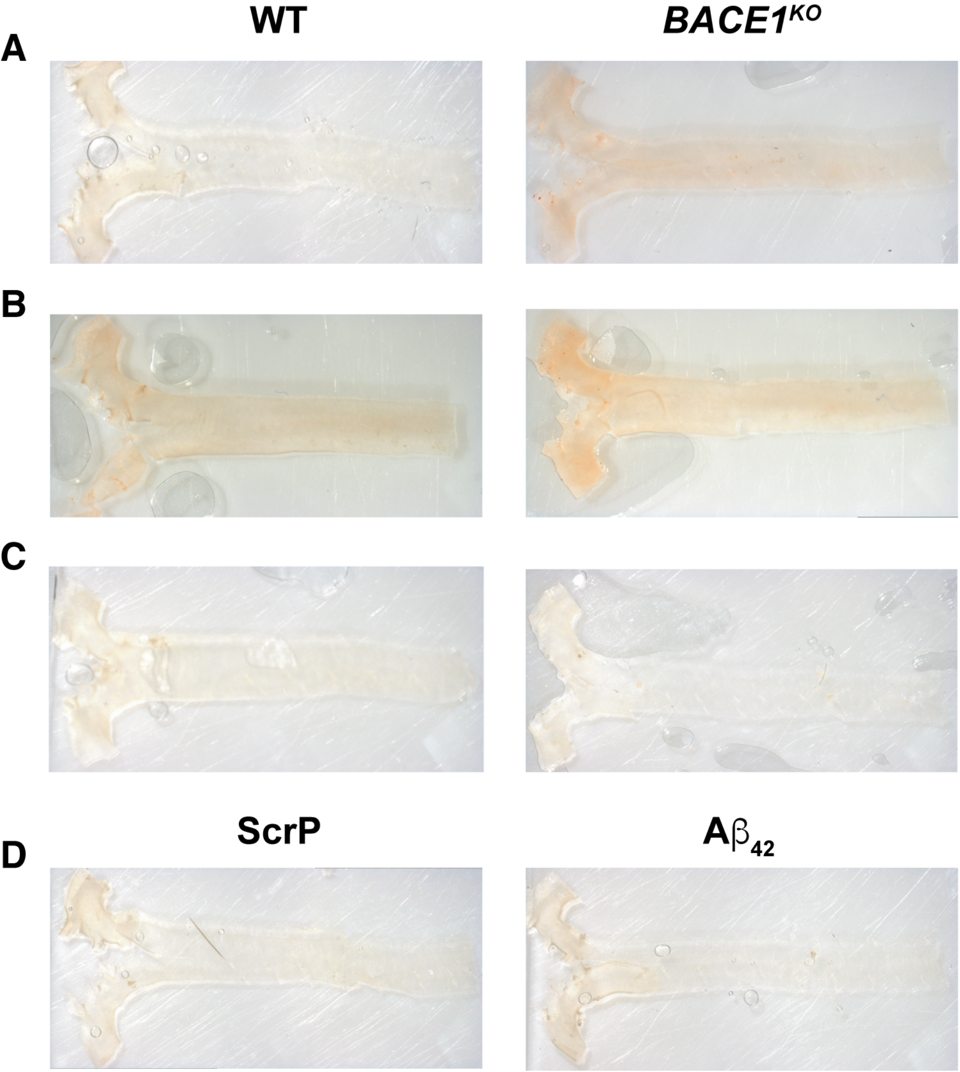

Supplemental Figure 5. Lack of lipid deposition in aortic arch. Representative images of aortic arches stained with Oil Red O. No lipid deposition was detected in aortic arch of WT and *BACE1*^{KO} mice on (**A**) regular chow or after (**B**) 5 weeks or (**C**) 12 weeks on high fat diet. (**D**) Infusion of ScrP or $A\beta_{42}$ did not cause lipid deposition.

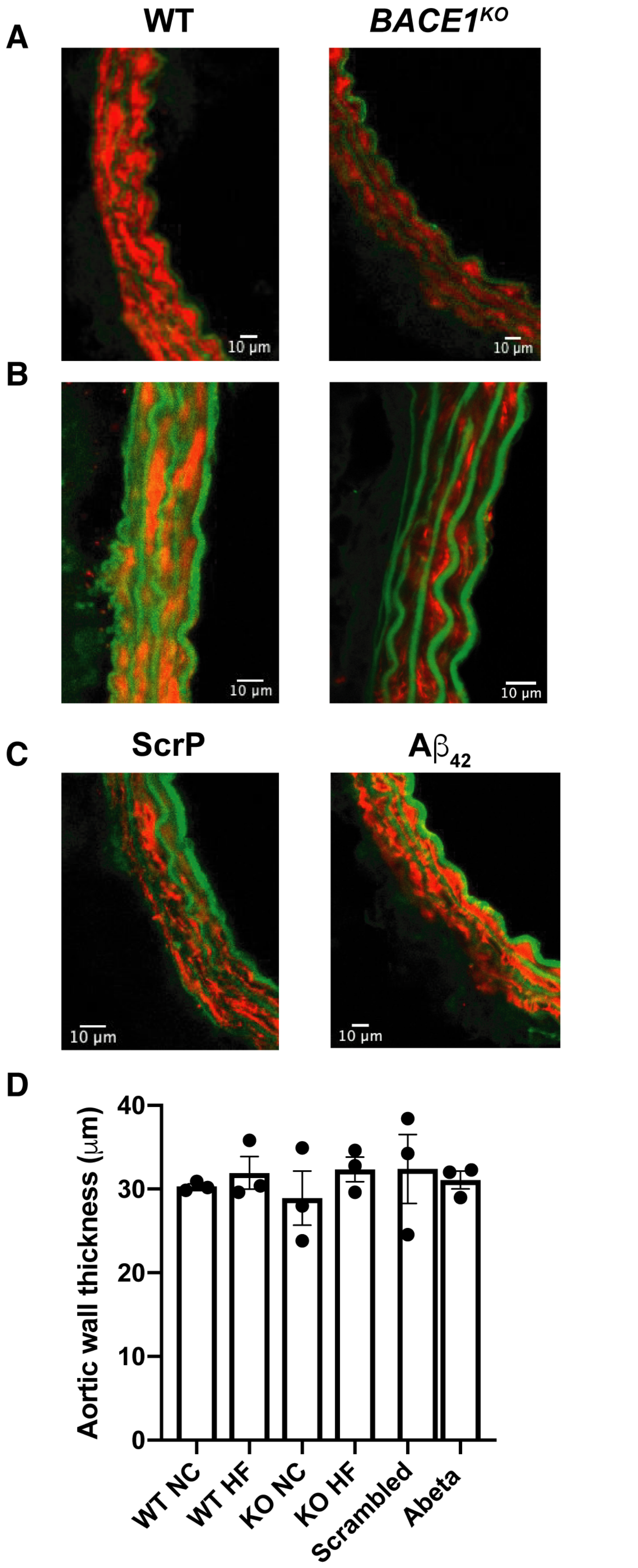

Supplemental Figure 6. Immunohistochemical analysis of aortic wall structure.
Representative images of peri-renal sections stained with Isolectin B4 (Green)and α-smooth muscle actin (Red) from WT and *BACE1*^{KO} mice on (**A**) RC-diet and (**B**) 12 weeks on high fat diet or (**C**) following infusion of ScrP or Aβ_{42}.(**D**) Aortic wall thickness is unchanged by these treatments, n = 3.

Supplemental Table 1.

Demographic characteristics of participants undergoing CABG surgery.

	Lean	Overweight	Obese
N	28	80	7
Age (years)	68.6 (10.8)	66.3 (9.9)	71.3 (7.2)
BMI (kg/m^2)	22.8 (2.0)	29.9 (2.3)	37.3 (2.6)
Diabetes (%)	14.3	5.7	42.8
Insulin (mIU/L)	7.4 (9.7)	10.9 (10.8)	11.1 (10.1)
Glucose (mg/dL)	97 (30.9)	109 (36.9)	125.7 (39.7)
HOMA-IR	1.9 (3.5)	2.0 (1.7)	3.6 (3.5)

Values are expressed as means with SD in brackets.

Supplemental Table 2.

(**A**) General composition of regular chow (RC) and high fat (HF) diets and (**B**) carbohydrate and fat composition of diets for mice.

A

Kcal composition	**RC (RM1)**	**HF (824053)**
Crude fat (%)	7.4	45
Crude Protein (%)	17.5	20
Carbohydrate (%)	75.1	35

B

Carbohydrate/Fat composition	**RC**	**HF**
Rice starch (% w/w)	45	28.3
Mixed sugars (% w/w)	4.5	-
Soya oil (% w/w)	2.71	4.3
Sucrose (% w/w)	-	10.5
Lard (% w/w)	-	17.9

Supplemental Table 3.

Source, catalogue number, species and dilution of antibodies used for immunoblots.

Primary Antibody	Species	Dilution	Source
Bace1	Rabbit	1:500 (IF)<br>1:3000 (IHC)	Genetex<br>(GTX113319)
CD31	Mouse	1:25 (IHC)	Abcam<br>(ab9498)
Smooth muscle actin<br>(SMA)	Mouse	1:1000 (IHC)	Abcam<br>(ab7817)
Smooth muscle actin<br>(SMA)	Rabbit	1:100 (IHC)	Abcam<br>(ab5694)
peNOS<br>(Ser1177)	Mouse	1:1000 (IB)	BD Transduction<br>(612392)
eNOS	Rabbit	1:1000 (IB)	Cell Signalling<br>(32027)
pPKB<br>(Ser473)	Rabbit	1:1000 (IB)	Cell Signalling<br>(9271)
PKB	Rabbit	1:1000 (IB)	Cell Signalling<br>(4691)
pAMPK<br>(Thr172)	Rabbit	1:1000 (IB)	Cell Signalling<br>(2535)
AMPK	Rabbit	1:1000 (IB)	Cell Signalling<br>(5832)
PKG	Rabbit	1:1000 (IB)	Cell Signalling<br>(3248)
pVASP<br>(Ser239)	Rabbit	1:1000 (IB)	Cell Signalling<br>(3114)
ICAM1	Mouse	1:1000 (IB)	R&D Systems<br>(AF796)
Actin	Rabbit	1:5000 (IB)	Sigma Aldrich<br>(A2066)
